# Breaking boundaries: role of the brain barriers in metastatic process

**DOI:** 10.1186/s12987-025-00618-z

**Published:** 2025-01-08

**Authors:** Nasim Izadi, Peter Solár, Klaudia Hašanová, Alemeh Zamani, Maryam Shahidian Akbar, Klára Mrázová, Martin Bartošík, Tomáš Kazda, Roman Hrstka, Marek Joukal

**Affiliations:** 1 Research Centre for Applied Molecular Oncology, Masaryk Memorial Cancer Institute, Zluty Kopec 7, 656 53 Brno, Czech Republic; 2 Department of Anatomy, Cellular and Molecular Neurobiology Research Group, Faculty of Medicine, Masaryk University, 625 00 Brno, Czech Republic; 3 Department of Neurosurgery, Faculty of Medicine, Masaryk University, St Anne University Hospital Brno, Pekařská 53, 656 91 Brno, Czech Republic

**Keywords:** Brain Metastasis, Blood–brain barrier, Blood-spinal cord barrier, Blood-cerebrospinal fluid barrier, Cancer

## Abstract

Brain metastases (BMs) are the most common intracranial tumors in adults and occur 3–10 times more frequently than primary brain tumors. Despite intensive multimodal therapies, including resection, radiotherapy, and chemotherapy, BMs are associated with poor prognosis and remain challenging to treat. BMs predominantly originate from primary lung (20–56%), breast (5–20%), and melanoma (7–16%) tumors, although they can arise from other cancer types less frequently. The metastatic cascade is a multistep process involving local invasion, intravasation into the bloodstream or lymphatic system, extravasation into normal tissue, and colonization of the distal site. After reaching the brain, circulating tumor cells (CTCs) breach the blood–brain barrier (BBB).

The selective permeability of the BBB poses a significant challenge for therapeutic compounds, limiting the treatment efficacy of BMs. Understanding the mechanisms of tumor cell interactions with the BBB is crucial for the development of effective treatments. This review provides an in-depth analysis of the brain barriers, including the BBB, blood-spinal cord barrier, blood-meningeal barrier, blood-arachnoid barrier, and blood-cerebrospinal fluid barrier. It explores the molecular and cellular components of these barriers and their roles in brain metastasis, highlighting the importance of this knowledge for identifying druggable targets to prevent or limit BM formation.

## Background

Metastasis into the brain poses a challenging task for tumor cells to overcome specialized brain barriers that carefully regulate the flow of substances, such as nutrients, cells, or drugs, between the bloodstream and brain tissue. The mechanisms developed by tumor cells are quite sophisticated but clearly successful because brain metastases (BM) represent the most prevalent intracranial tumors in adults, appearing up to 3–10 times more often than primary brain tumors. [1]. BMs are associated with poor prognosis and are difficult to treat even after intensive multimodal therapy, including resection, radiotherapy, and chemotherapy [2, 3]. They are indicated by symptoms that include seizures, loss of motor and sensory functions, or cranial neuropathies and are usually identified by imaging. One study showed that BMs mainly originate from lung (43.2%), breast (15.7%), and melanoma (16.4%) primary tumors [4] but may appear less frequently due to other cancer types.

Several molecular mechanisms contribute to brain colonization by metastatic tumor cells. In general, the metastatic cascade is a multistep process involving (a) local invasion into nearby tissue, (b) intravasation, that is, entry into the bloodstream or lymphatic system, (c) vascular travel, (d) extravasation, that is, the exit of circulating tumor cells (CTCs) from the bloodstream into normal tissue, and (e) colonization of the distal site [5]. To migrate, cells usually undergo epithelial-mesenchymal transition (EMT), a complex process in which cancer cells shed their epithelial characteristics, become less differentiated, and have more aggressive and stem cell-like characteristics [6, 7]. EMT occurs during local invasion and intravasation. It is regarded as a key biological process in which polarized epithelial cells lose their interaction with the basement membrane due to myriad biochemical changes. These newly formed mesenchymal cells have the capacity to migrate and invade their surroundings and possess the ability to overcome apoptosis [8]. EMT is characterized by decreased expression of E-cadherin, increased expression of N-cadherin [9], and downregulation of β-catenin, a key player in cell junction formation [10]. Once metastatic cells reach the distal site, a reverse process to EMT, termed mesenchymal-to-epithelial transition (MET), occurs [11]. Regardless of the primary cell type [12], MET constitutes an initial stage in the adaptation of CTCs to their new microenvironment during colonization. During the MET process, the expression of genes that were previously attenuated during EMT is reactivated, leading to observable changes in tumor cell morphology [13]. This transient phase of cellular reprogramming is associated with increased coexpression of epithelial markers, such as E-cadherin, and mesenchymal markers, such as vimentin [12, 14]. In the case of BMs, CTCs must extravasate and cross the blood–brain barrier (BBB) by breaching the tight junctions (TJs) between endothelial cells in brain capillaries. This involves the secretion of proteases and the activation of pathways that disrupt the integrity of the BBB.

The BBB is perhaps the major reason for the high incidence of BMs since many therapeutic compounds cannot penetrate the barrier, lowering treatment efficiency [15]. Since BMs represent a great therapeutic challenge, it is crucial to better understand the mechanisms of the interaction of tumor cells with the BBB to find druggable targets to prevent or at least limit BM formation. Hence, in this review, we provide a thorough analysis of intricate brain barriers and the complex mechanisms involved in the metastasis of tumor cells into the brain to understand the complex interplay of brain barriers and the intricate mechanisms governing cancer metastasis, offering potential insights for therapeutic interventions and further research endeavors in the field.

## Brain barriers and their permeability

**Fig. 1:**
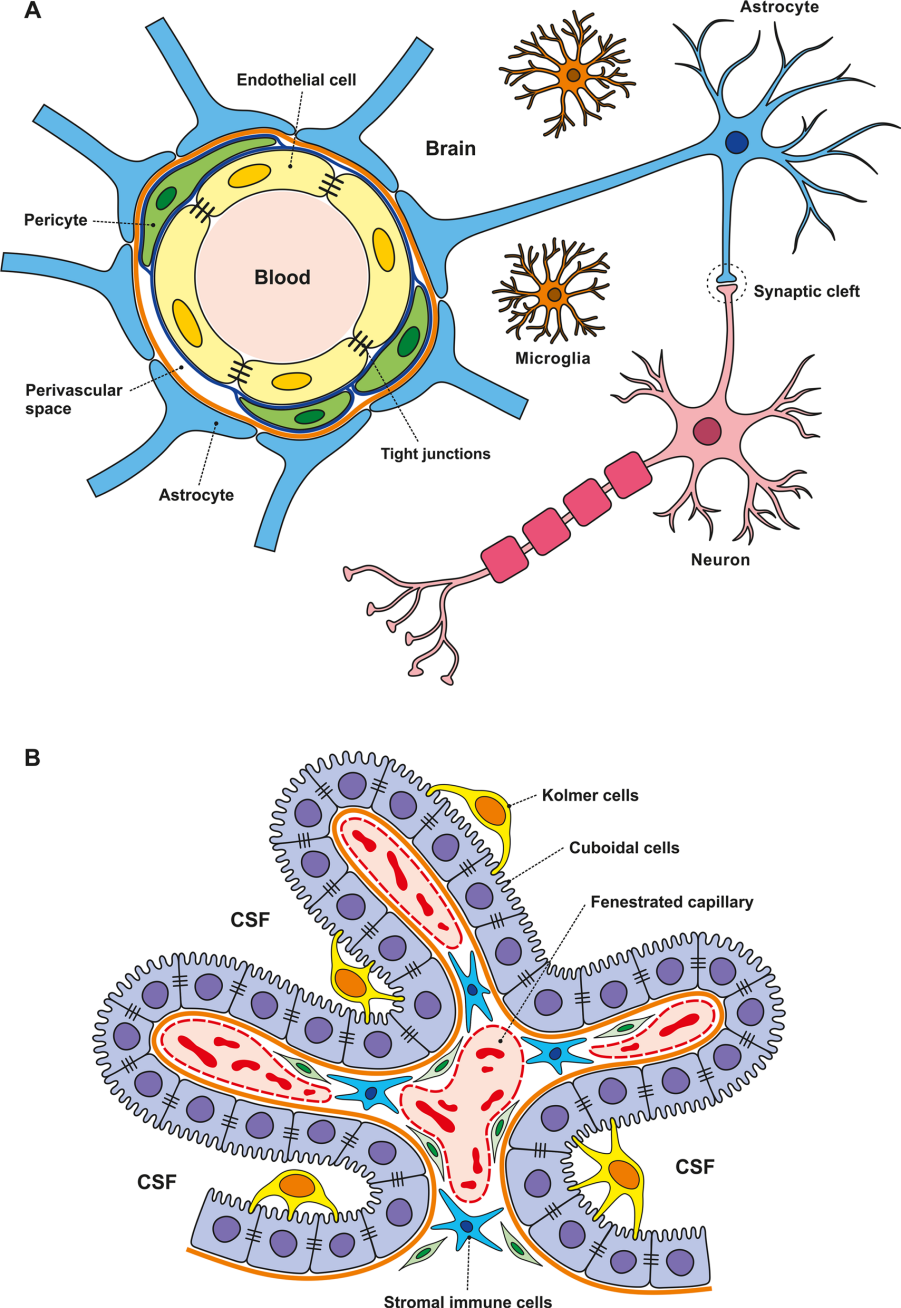
Schematic illustration of key central nervous system barriers: Blood–Brain Barrier (BBB) and Blood-Cerebrospinal Fluid Barrier (B-CSF barrier). **A** Schematic representation of the BBB, illustrating the interaction between brain endothelial cells, pericytes, and astrocytic end-feet that collectively form the barrier. **B** Illustration of the CP, a vascular structure in the brain ventricles composed of a stromal core and a single-layer cuboidal epithelium forming the B-cerebrospinal fluid (CSF) barrier

The central nervous system (CNS), comprising the brain and spinal cord, is protected by specialized interfaces that selectively permit the exchange of nutrients, ions, various other molecules, including drugs and contrast agents for brain imaging, and even whole cells between the brain and blood [16]. This allows for the precise control of CNS homeostasis, which is critical for proper neuronal function. These major brain barriers or interfaces, including their structure and role in the migration of metastatic cells into the CNS are discussed. The BBB and blood-cerebrospinal fluid (B-CSF) barrier play a major role in the migration of metastatic cells to the brain and are discussed in detail (Fig. 1A-B; Fig. 4; Fig. 5 and Fig. 7). Much less information is known about the migration of metastatic cells to the CNS via the blood-arachnoid barrier (BAB) (Fig. 6). Other barriers in the CNS include the blood-spinal cord barrier (BSCB), however, the role of this barrier in the spread of metastases remains unknown. The microenvironment of the brain parenchyma is tightly regulated by the BBB, the most selective physical barrier in the CNS. The unique properties of the BBB are manifested by the molecular components (junctional proteins and transporters) of the endothelial cells (ECs) of blood vessels by which the CNS is vascularized. The movement of ions, molecules, and cells between the blood and brain is also facilitated by interactions between ECs and different vascular, immune, and neuronal cells, known as the neurovascular unit (NVU) [17–20].

The major difference between peripheral metastasis and brain metastasis lies in the unique challenges that metastatic tumor cells face when attempting to enter and survive in the brain environment. The BBB plays a significant role in this difference by acting as a selective barrier, which interacts with tumor cells and factors from the tumor microenvironment in ways that do not occur in peripheral metastasis. In the following section, we will discuss the molecular components, cellular components, and signaling pathways involved in the interaction between metastatic cells and the BBB, highlighting how these mechanisms contribute to the establishment and progression of brain metastasis.

### Molecular components of the BBB

The nonfenestrated structure of blood vessels in the brain formed by junctional proteins between ECs results in extremely limited paracellular movement. It allows only hydrophobic molecules of a relatively small size to cross the BBB. Junctional proteins include tight junction (TJ), adherens junction (AJ), and gap junction (GJ) proteins. The structure and function of junctional proteins have been extensively reviewed [21–24]. Briefly, TJs situated on the apical membrane of ECs consist of transmembrane proteins (claudin and occludin) that are connected to the cytoskeleton by cytoplasmic proteins (zonula occludens proteins; ZO). In addition, the expression profiles of the transporter proteins of ECs are not identical on the luminal and abluminal surfaces, which results in the formation of a highly polarized environment in ECs. Therefore, it allows distinct transport properties, and delivery of essential nutrients across the BBB. The transporters of ECs and their role in brain homeostasis have been well studied [17, 19, 25–30].

### Cellular components of the neurovascular unit

The NVU comprises ECs, mural cells (vascular smooth muscle cells and pericytes), immune cells, glial cells (astrocytes), and neurons [31]. Mural cells are in immediate contact with ECs on their abluminal side, thus regulating blood flow. Pericytes are embedded in the basement membrane and have the ability to contract and control diameter [17, 26]. The basement membrane, formed by the extracellular matrix, covers the vascular tube from both the luminal and abluminal sides. The luminal side is covered by ECs and pericytes, whereas the abluminal side is wrapped by astroglial endfeet [17, 18]. Pericytes are also involved in maintaining the stability of microcirculation through the angiogenic molecules and growth factors [32]. Even though pericytes are separated from ECs by the basement membrane, pericytes processes communicate with ECs through the synapse-like peg-socket contacts and thereby affect microcirculation [26]. Other important functions of pericytes in maintaining BBB homeostasis include immune or phagocytic functions stem cell potential, angiogenesis as well as participation in the development of the BBB [31, 33, 34].

Glial cells, mainly astrocytes, play significant and complex roles in BBB permeability via polarized cellular processes (endfeet), which ensheathe either neuronal processes or blood vessels. Therefore, a cellular link is formed between the neuronal circuit and blood vessels, enabling blood flow regulation in response to neuronal activity [17]. In addition, the expression of different proteins, such as aquaporin-4 in astrocytes, is critical for regulating the BBB [18].

### Blood-spinal cord barrier

The BSCB, a functional equivalent of the BBB, represents the interface between the spinal cord and the systemic circulation, therefore, serves to protect the spinal cord microenvironment [35–37]. Structurally, the barrier is composed of ECs joined by TJs and covered by a continuous basement membrane on the basolateral side. Like the BBB, the BSCB is supported by astrocyte endfeet expressing aquaporin 4, a key transporter that mediates water absorption [35]. ECs restrict the passage of molecules from the bloodstream into the spinal cord. Like the BBB, pericytes are crucial for the maintenance of the BSCB. However, fewer pericytes were observed in the BSCB. The different expression profiles of TJ proteins and transporters of ECs between the BSCB and BBB cause a relatively higher permeability of the BSCB. For example, lower expression of occludin and ZO-1, insulin receptor (INSR), low-density lipoprotein receptor-related protein 1 (LRP1), and glucose transporter 1 (GLUT1) in the BSCB has been reported [36–38].

### Blood-arachnoid barrier

The meninges, consisting of three layers of connective tissue, structurally protect the CNS by securing the brain to the skull, limiting lateral movement, and reducing the risk of injury to the brain and spinal cord. The outermost layer, the dura mater, is formed by two epithelial layers of dense collagen fibers and contains fenestrated vessels, allowing the transport of substances to the dura mater. Different immune cell types, including macrophages, T and B cells, and neutrophils, are present at this site. The leptomeningeal layer, composed of the arachnoid and pia mater, forms the innermost layer where the CSF fills the subarachnoid space. The arachnoid epithelial cells joined by TJs form the BAB, preventing the passage of cells and molecules into the CNS and the passage of CSF from the subarachnoid space into the dura mater. The inner layer of the arachnoid mater is connected to the pia mater by a layer of leptomeningeal cells linked by gap junctions and serves as a semipermeable membrane for solutes [39]. The absence of pericytes and astrocytic endfeet distinguishes BAB from the BBB. The greater expression of intercellular adhesion molecule 1 (ICAM-1) in the BAB than in the BBB makes the barrier more permissive for immune cells to circulate within meningeal spaces even under physiological conditions and as an entry site for lymphocytes and myeloid cells during inflammation [39, 40].

The subarachnoid space contains approximately 80% of the total CSF volume, and the middle layer of the meninges (arachnoid membrane), also called the blood-arachnoid barrier, covers the CSF in this space. The BAB separating the CSF and circulating blood consists of multilayered epithelium with cells joined by TJs. It is characterized by a continuous basal lamina on its inner surface, facing the innermost collagenous part of the arachnoid [41, 42].

The expression of multiple transporters in BAB suggests its importance in eliminating organic anions and neurotoxin cations from the CNS and regulating the passage of molecules between the CSF and fenestrated blood capillaries in the dura mater. It has been shown that transporter abundance in the BAB differs from those in the BBB, which separates the blood and the interstitium of the brain and B-CSF barrier located in the choroid plexus of the brain ventricles [43]. The expression of protein transporters in BAB varies between species [44, 45]. Some of the protein transporters such as OCT3, MCT4 and OATP1A2 have been found in BAB only in humans. On the other hand, efflux transporter MRP3 was found in BAB of pigs and dogs, not in human [44]. The transporter system in porcine BAB contributes to a greater overall transfer of substances compared to the B-CSF barrier. This is probably due to the larger area of the BAB and, thus, higher absolute expression of the transporters than in the B-CSF barrier [45]. For instance, organic anion transporter (OAT)1 and OAT3 and drug-metabolizing enzymes (COMT, GSTP1, and CES1) have been shown to be abundantly expressed in the BAB, while they have been detected at low levels in the BBB and B-CSF barrier [44].

Takeuchi et al. demonstrated that drug transporter expression profiles differ in different regions of the BAB [43]. However, this was not comparable to that at the BSCB. As such, multidrug resistance 1 (MDR1) expressed in both the BAB and BSCB was shown to be lower in the lumbar region at the BSCB than at the BAB. In contrast, it was greater in the cervical region of the BSCB. Moreover, breast cancer resistance protein (BCRP), a restricting transporter of various drugs into the CNS, is expressed at higher levels in the BSCB than in the BAB, with higher expression in the cervical portion [42, 46]. Furthermore, the weaker integrity of either the BAB or BSCB in the lumbar cord was suggested to be due to the leakage of proteins from the blood. Takeuchi et al. reported that although the expression of claudin-11 at the BAB in the cervical and lumbar regions was comparable, claudin-11 expression was lower in the lumbar region at the BSCB.

### Blood-cerebrospinal fluid barrier

The choroid plexus (CP), located at the brain ventricles, plays a significant role in the maintenance of CNS homeostasis. The CP comprises highly vascularized stroma, connective tissue, and epithelial cells. The fenestrated capillaries formed by endothelial cells allow for the free entry of cells and molecules into the stroma. However, tightly joined epithelial cells strictly control the passage of substances into the CSF of the brain to form the B-CSF barrier. On the ventricular side of epithelial cells, epiplexus (Kolmer) cells exhibit phagocytic activity [47]. The epithelial cells of the B-CSF barrier express a complex system of junctional and transporter proteins that are fundamental to barrier permeability and integrity. Compared with the BBB, the B-CSF barrier is known to be “leaky,” which is also manifested by the fact that, on brain imaging scans, they become enhanced after the administration of contrast agents. This is believed to be due to the distinct TJ levels between the two barriers [48].

The components of the B-CSF barrier are innervated by the autonomic nervous system, which involves the superior cervical ganglion and parasympathetic fibers of the glossopharyngeal and vagus nerves. Parasympathetic stimulation decreases Na^+^-K^+^ adenosine triphosphate-hydrolyzing enzyme (ATPase) activity, which leads to decreased CSF secretion [47]. The wide range of transporters involved in CP activity leads to semipermeable characteristics of the B-CSF barrier. This is critical for the exchange of metabolites and xenobiotics between the blood and CSF [47].

The epithelial cells of the CP are connected via different types of cell junctions, which regulate the permeability and integrity of the B-CSF barrier. One of the most important cell junctions is the tight junctions (TJs) of the apical epithelial cells of the CP, which are responsible for regulating the paracellular diffusion of water-soluble molecules through this barrier. Moreover, TJs maintain electrical resistance across the epithelial layer of the CP. The main transmembrane molecules are occludin and claudin [1–6, 9–12, 19, 22], while the submembrane molecules are zonula occludens (ZO), i.e., ZO-1, ZO-2 and ZO-3. The basolateral epithelial cells of the CP are also connected via AJs, which are linked to cytoskeletal proteins. Among epithelial cells, intracellular protein channels represented by GJs, formed by two hemichannels composed of six connexin proteins, have been identified. GJs mediate intercellular communication and exchange of metabolites and electrolytes [47].

## Brain metastases

The generally accepted ‘seed and soil’ hypothesis describes a cancer cell as a ‘seed’ implanted into a suitable environment, i.e., a ‘soil’ [49]. As we mentioned, metastasis is preceded by the EMT process, whereby cancer cells acquire the ability to migrate and behave more aggressively. Metastasis is affected by the qualities of the ‘seed’ as well as the ‘soil’ [50]*.* Cancer cells may anchor at a distant site, become dormant, and create metastases after a certain period [51]. The state of dormancy is also influenced by microenvironmental conditions in various organs [50]. The metastatic process further depends on the type of molecules [50] and extracellular vesicles (EVs), mostly exosomes, released by tumor cells [52].

Exosomes, which can be found in blood [53], urine [54], saliva [55], or cerebrospinal fluid [56], mediate cell–cell communication [57] and transport numerous types of proteins, lipids, and nucleic acids [58, 59]. Compared with healthy cells, tumor cells release more exosomes [60]. These are known as tumor-derived exosomes (TDEs) and play essential roles in altering the cancer cell microenvironment [61, 62] and all stages of metastasis [63].

### Exosomal shaping of the microenvironment

**Fig. 2:**
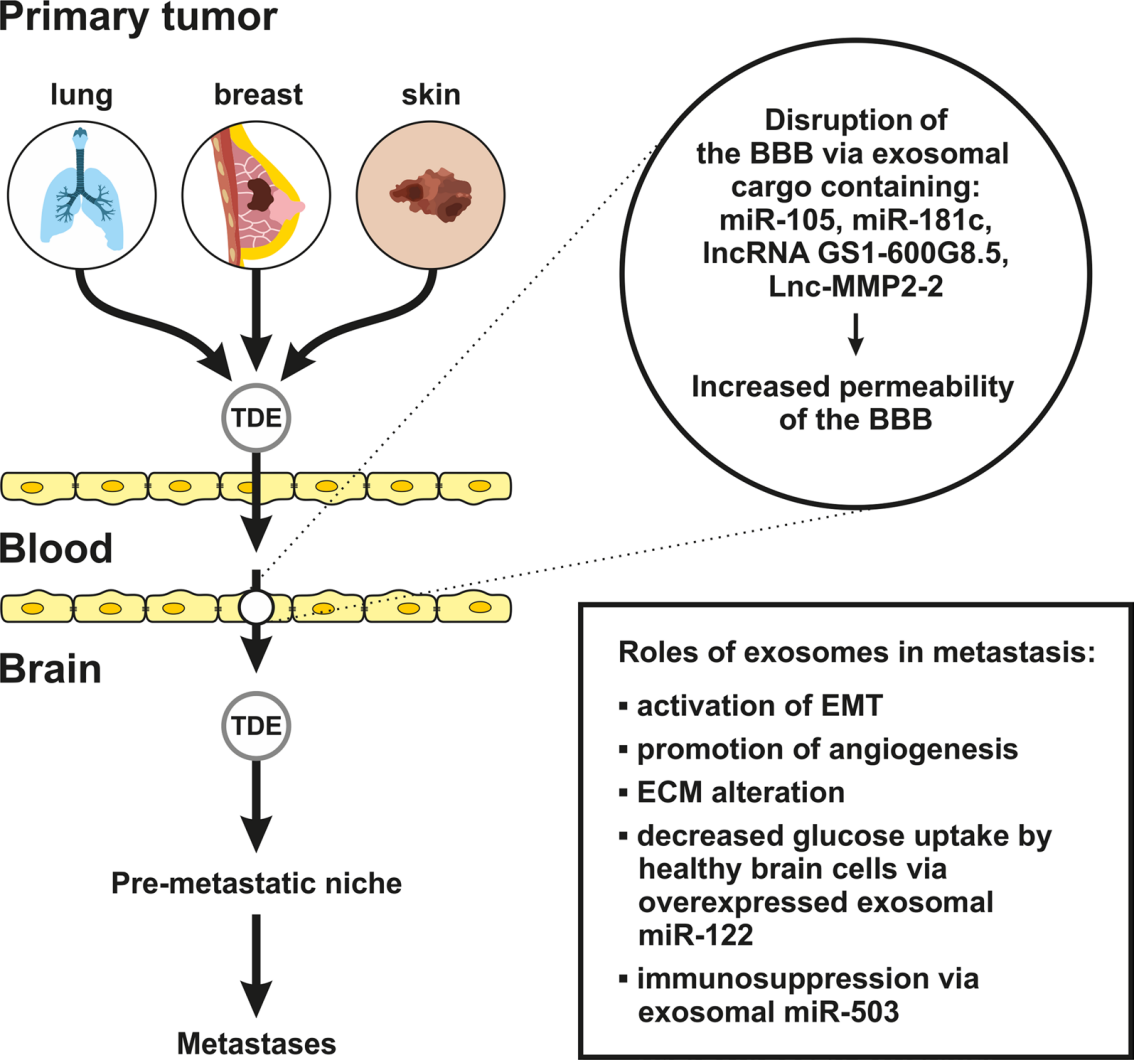
Tumor microenvironment and exosomes in brain metastasis: Molecular mechanisms. Tumor cells and exosomes originating from primary tumors can disrupt the BBB (blood–brain barrier) via their contents. Exosomes carry microRNAs (such as miR-105 and miR-181c), whose involvement leads to increased permeability of the blood–brain barrier (BBB). LncRNA GS1-600G8.5 lowers the expression of ZO-1, Claudin-5, and N-Cadherin. Lnc-Matrix metallopeptidases (MMP)2–2, as part of the exosomal cargo, also disrupt tight junctions, thereby increasing the permeability of the BBB. This promotes intracranial metastasis of tumor cells. Exosomes interact with microglia in the brain and help establish pre-metastatic niches. Furthermore, exosomes promote the proliferation of the BM (brain metastases), alter the immune microenvironment, regulate the stability of tumor cells, and can be used as diagnostic and prognostic biomarkers

Exosomes can modify the primary microenvironment, primary soil [64], and distant soil [65]. They participate in the activation of EMT by acting as messengers and sending signals from the tumor to recipient cells, thus causing changes in the properties of the recipient cells and their microenvironment [8] (Fig. 2). TDEs also affect the extracellular matrix (ECM) [66]. It has been proposed that matrix metalloproteinases (MMPs) might be integrated into exosomal cargo [67]. Migration and invasion have been linked to MMP2 in lung cancer-derived exosomes [68]. TDEs cause ECM degradation via MMPs [69], thereby stimulating cell motility and influencing the formation of a pre-metastatic niche [70]. Exosomal micro RNAs (miRs) are responsible for the destruction of the BBB integrity. For instance, miR-105 originating in breast cancer cells downregulates ZO-1 tight junctions [71]. Similarly, exosomal miR-181c causes downregulation of phosphoinositide-dependent protein kinase 1, which subsequently leads to inhibition of actinin phosphorylation, cofilin activation, and changes in actin conformation. As a result, tight connections of the BBB are destroyed [72]. Moreover, metastatic cells use exosomal miRs to promote cancer cell proliferation and immunosuppression. Metastatic cell proliferation is enhanced by exosomal miR-122 which increases glucose availability to cancer cells through the downregulation of PKM2 and GLUT1 in niche cells [73]. Following the transfer of metastatic cells through the BBB into the brain tissue, the survival of these cells is supported by exosomal miRs-induced immunosuppression. An example is the conversion of microglia to anti-inflammatory M2 phenotype by exosomal miR-503 [74].

TDE cargo also contributes to inflammatory processes; for example, Annexin A2 in TDEs released from breast cancer tissue is responsible for the activation of several signaling pathways, including the p38 mitogen-activated protein kinase (MAPK), nuclear factor κB (NF-κB), and signal transducer and activator of transcription 3 (STAT3) pathways within the cell. This leads to increased concentrations of interleukin (IL)-6 and tumor necrosis factor α (TNF-α), which results in the creation of a pre-metastatic inflammatory microenvironment at distant sites, mainly the lungs and brain [75]. The characteristics of the secondary microenvironment, i.e., the ‘soil’ in distant organs, are thus critical in the development of cancer metastasis [50].

However, tumor cells at the primary site are responsible for the release of exosomes. A classic example is the differentiation of stromal cells into cancer-associated fibroblasts (CAFs), which are triggered by exosomes produced by various tumor cells, such as those of the stomach, colon, and bladder, among others. Transformation into CAFs may involve normal (healthy) fibroblasts in primary tumors, cells in distant metastatic tissues, or other types of cells, such as adipocytes and endothelial or mesenchymal stem cells [76, 77].

ECM of the brain is quite different from other organs in several important ways. In most non-CNS tissues, ECM proteins like collagens, laminins, and fibronectin are widely distributed throughout the tissue. However, in the brain, these proteins are mainly found in association with the vascular basement membranes. In contrast, the major ECM components of normal brain parenchyma include proteoglycans, glycoproteins, and glycosaminoglycans, particularly heparan sulfate proteoglycans (HSPGs) and hyaluronic acid (HA) [78], which may lead to different ECM functions [79]. Stevens et al. demonstrated that the hyaluronan-mediated motility receptor (HMMR) is a key player in the process of lung cancer metastasis, particularly to the brain [80]. HMMR is an ECM receptor that binds to HA, a glycosaminoglycan that is often overproduced in the tumor microenvironment during inflammation and fibrosis. This connection is particularly relevant in lung adenocarcinoma (LUAD), where HMMR expression has been associated with aggressive tumor behavior and poor prognosis [80].

### Organotropism and tumor-derived exosomes

#### Role of exosomes in pre-metastatic niche formation

The mechanism of organ-specific metastasis is complex and involves a variety of mechanisms, including EVs, which may influence the interaction between tumor cells and distant sites [65]. Hoshino et al. showed that the uptake of exosomes in the brain could create a pre-metastatic microenvironment for tumor metastasis and demonstrated that the integrin expression profiles of circulating plasma exosomes isolated from cancer patients could be used as prognostic factors to predict sites of future metastasis [81].

#### Organ-specific properties and genetic adaptation

In contrast to the BBB, the fenestrated sinusoidal endothelium allows extravasating cells to penetrate more easily [82]. Certain types of metastatic tumor cells have a greater ability to penetrate the BBB than other types do. These are mainly tumor cells from breast cancer, lung cancer, and melanoma, but also renal and colorectal cancer [79]. During the initial stages of BM, metastatic cells operate in a hypoxic environment with a lack of glucose [83–85] and thus must express a certain set of genes [79]. Unlike primary tumor cells, tumor cells in brain metastases may harbor mutations in genes such as *ERBB2*, *BRAF*, *MYC*, and *BRCA2* [86]. Moreover, BMs may be supported by exosomal miRs that alter the microenvironment to become more favorable for BM formation [71].

#### Exosomal contribution to tumor progression

The gene composition of the primary tumor is essential in the context of organotropism. In the case of non-small cell lung cancer (NSCLC), mutations in *the EGFR* or *ALK* genes lead to enhanced metastasis to the CNS in approximately 50% of patients [87]. Moreover, the gene composition of a primary tumor influences which areas of the brain are affected [88]. In breast cancer, the expression of the NAD^+^-dependent deacetylase Sirtuin 1 (SIRT1) is decreased, which subsequently leads to increased secretion of exosomes [89]. These exosomes then contribute to tumor growth, the migration of cancer cells, the degradation of the ECM, and higher vascular permeability and metastasis to distant sites. Exosomes derived from lung cancer cells can activate EMT, which promotes migration, invasion, proliferation [90], and angiogenesis [91], hence supporting metastasis. Moreover, they contribute to immune escape, thereby facilitating the progression of lung cancer [92]. In melanoma, exosomes can influence the invasion and motility of cancer cells [93]. Metastasis is also facilitated by exosome-mediated immunosuppression [94] and vascular leakage [72].

### Involvement of the immune system

Neuroinflammation precedes BM formation [95]. Pro-inflammatory molecules originating from astrocytes intensified tumor growth in a model of melanoma-derived BM [96]. Similarly, it seems that tumor growth is promoted by glial cells infiltrating BMs by producing pro-inflammatory molecules [79].

The tumor immune microenvironment (TIME) is involved in the entire process of carcinogenesis [97]. It varies slightly from organ to organ and encompasses different types of immune cells, including granulocytes, lymphocytes, monocytes/macrophages, and dendritic cells [98]. Usually, immune cells cooperate to eliminate tumors. However, this ability can be silenced or even reversed so that immune cells can suddenly support tumor progression [97]. Specific mutations in tumor cells are responsible for the secretion of chemokines that draw immune cells to the tumor [99].

Tumor cells communicate with immune cells and dictate their behavior to a certain extent. For example, macrophages, monocytes, neutrophils, and other myeloid populations are able to subdue the cytotoxic characteristics of lymphocytes [97, 100, 101]. Immune cells, mainly macrophages, may stimulate EMT [102]; however, macrophages also work in the opposite manner by encouraging the reverse MET process at the metastatic site [103]. Moreover, experiments with BALB/c mice have shown that NK cells are important for protection from colonization by metastasis or that the loss of NK cells leads to the progression of metastasis [104]. Perforin is a major contributor to NK cell control during tumor metastasis [104]. Higher cytotoxic activity of NK cells or increased expression of NK cell activation receptors indicates a better prognosis for patients at risk of metastatic disease [105].

Hypoxic conditions in solid tumors significantly affect immune cells. Activation of HIF-1α-dependent transcriptional changes in immune cells results in adjustment of their function [106], including immunosuppression in macrophages [107]. Another consequence of a hypoxic state is increased production of monocyte recruitment factors [108], promotion of glycolysis in cancer cells, and the higher production of lactic acid [109].

### Metastasis of lung cancer to brain tissue

Lung cancer is one of the most common causes of cancer-related death worldwide and the leading cause of BM [110–112]. The frequency of BM is markedly greater in patients with lung cancer than in those with other common cancers [2]. Small cell lung cancer (SCLC) and NSCLC are two main histological cancer subtypes that frequently spread to the brain [111, 113]. Approximately 10% of all SCLC patients develop BM [114]. In contrast, patients with NSCLC, which can be further subdivided into squamous cell carcinoma, adenocarcinoma, and large cell carcinoma types, have 20, 18, and 11% metastasis rates to the brain, respectively [115, 116]. Typically, lung cancer presents with multiple BMs.

#### Genes involved in BMs derived from lung cancer

In NSCLC, the most common mutations occur in the *TP53* gene encoding the tumor suppressor p53 (in almost 50% of cases), and these alterations are associated with a worse prognosis and may be relatively more resistant to chemotherapy and radiation [117]. TP53 regulates vital cellular processes, including DNA repair, cell cycle control, and apoptosis. The involvement of the *TP53* gene in lung cancer pathogenesis is underscored by frequent loss of heterozygosity (LOH) at its locus on chromosome 17p13, particularly in SCLC and squamous cell carcinoma [118]. *TP53* mutations, which occur early in tumorigenesis, persist throughout tumor progression and metastasis. These mutations, along with increased *TP53* expression, are correlated with poor survival and resistance to therapy in NSCLC [119]. Various studies have highlighted TP53 as a prognostic marker, with aberrant *TP53* status indicating aggressive disease and shorter survival, particularly in patients with adenocarcinomas [120]. Additionally, the *TP53* status influences the response to adjuvant chemotherapy [117], with greater benefits in TP53 + patients. Although *TP53* alterations are associated with poor prognosis, definitive evidence supporting their role in individual patient management remains elusive. Nonetheless, understanding the importance of TP53 in NSCLC underscores its potential as a therapeutic target and prognostic indicator [117].

Additional gene mutations implicated in NSCLC include those of *EGFR* and *KRAS*, the mutation rates of which depend on ethnicity, histology, and smoking status [121, 122]. EGFR, also known as ErbB-1 or HER1, is a transmembrane glycoprotein that belongs to the ErbB family of tyrosine kinase (TK) receptors, which also includes the homologous receptors HER2, HER3, and HER4 [123, 124]. EGFR and its family members contribute to several complex signaling cascades, including growth, differentiation, adhesion, migration, and survival of cancer cells [123, 125]. Ligand binding triggers hetero or homodimerization, trans-autophosphorylation, and the subsequent activation of various kinase cascades. These include the RAS-RAF-MEK-MAPK, PI3K-AKT-mTOR, PLC-γ-Ca2 + /PKC, and JAK-STAT3 pathways, which collectively promote the expression of genes associated with the progression of BMs [126].

EGFR overexpression is often observed in many solid tumors, such as breast cancer, head and neck cancer, NSCLC, renal cancer, ovarian cancer, and colon cancer [127]. Several studies have reported *EGFR* mutations in BMs arising from NSCLC [115, 122, 128, 129]. These studies revealed that patients with BM were more likely to have primary tumors with *EGFR* mutations; in particular, the prevalence of *EGFR* mutations in East Asian cohorts is high, found in 44–63% of BM cases in this population [115, 130, 131]. In a study by Eichler et al., 93 patients with NSCLC were clinically selected for *EGFR* mutation screening. Among these patients, 41 (44%) developed BM, indicating the importance of the *EGFR* mutation status as a prognostic factor [130]. TK domain mutations in *EGFR* occur mainly in exons 18–21. The most frequently reported mutations include exon 19 deletions and point mutations in exon 21, such as L858R, L861Q, and S768I. Additionally, a case report detailed the G719X mutation in exon 18 [115, 130, 132, 133].Sun et al. reported that copy number variations in EGFR were also associated with BMs. They reported an increase in *EGFR* copy numbers not only in primary NSCLC (62%) but also in corresponding brain metastases (64%) [124, 131]. Similarly, other studies revealed a gain of chromosome 7p, which could be related to *EGFR* amplification and, thus, a greater number of BMs [132].

KRAS has emerged as another extensively explored target in lung cancer-associated BMs. The *KRAS* oncogene encodes a small GTPase transductor protein bearing the same name [134]. Among human cancers, *KRAS* is one of the most frequently mutated oncogenes, occurring in 95% of pancreatic, 50% of colorectal, and 32% of lung adenocarcinomas [135]. KRAS, along with two family members, neuroblastoma RAS viral oncogene homolog (NRAS) and Harvey rat sarcoma virus (HRAS), functions as a transducer of signals that activate growth factor receptors [135]. Although *KRAS* comprises six exons, the majority of mutations are concentrated in codons 12, 13, 61, and 146. In NSCLC, G12C is the most prevalent mutation, accounting for almost half of all cases, followed by G12V and G12D [136]. Most studies have indicated that the diagnosis of BM in patients with *KRAS* mutations is lower than in patients with *EGFR* mutations. In a study involving 144 patients, Tomasini et al. compared *KRAS* and *EGFR* and reported that BMs associated with *EGFR* mutations were more common (87%) than those associated with *KRAS* mutations (55%) [137]. In a different study, Vassella et al. reported that 58% of 111 patients with KRAS mutations had brain metastasis. Additionally, they identified 14 patients with alterations in the *EGFR* pathway, including *KRAS* alterations associated with BM. The G12C mutation in *KRAS* was the most frequently detected mutation, occurring in 26% of the patients. Moreover, the *KRAS* G12C and G13C variants are notably enriched in patients with BM [123].

**Fig. 3:**
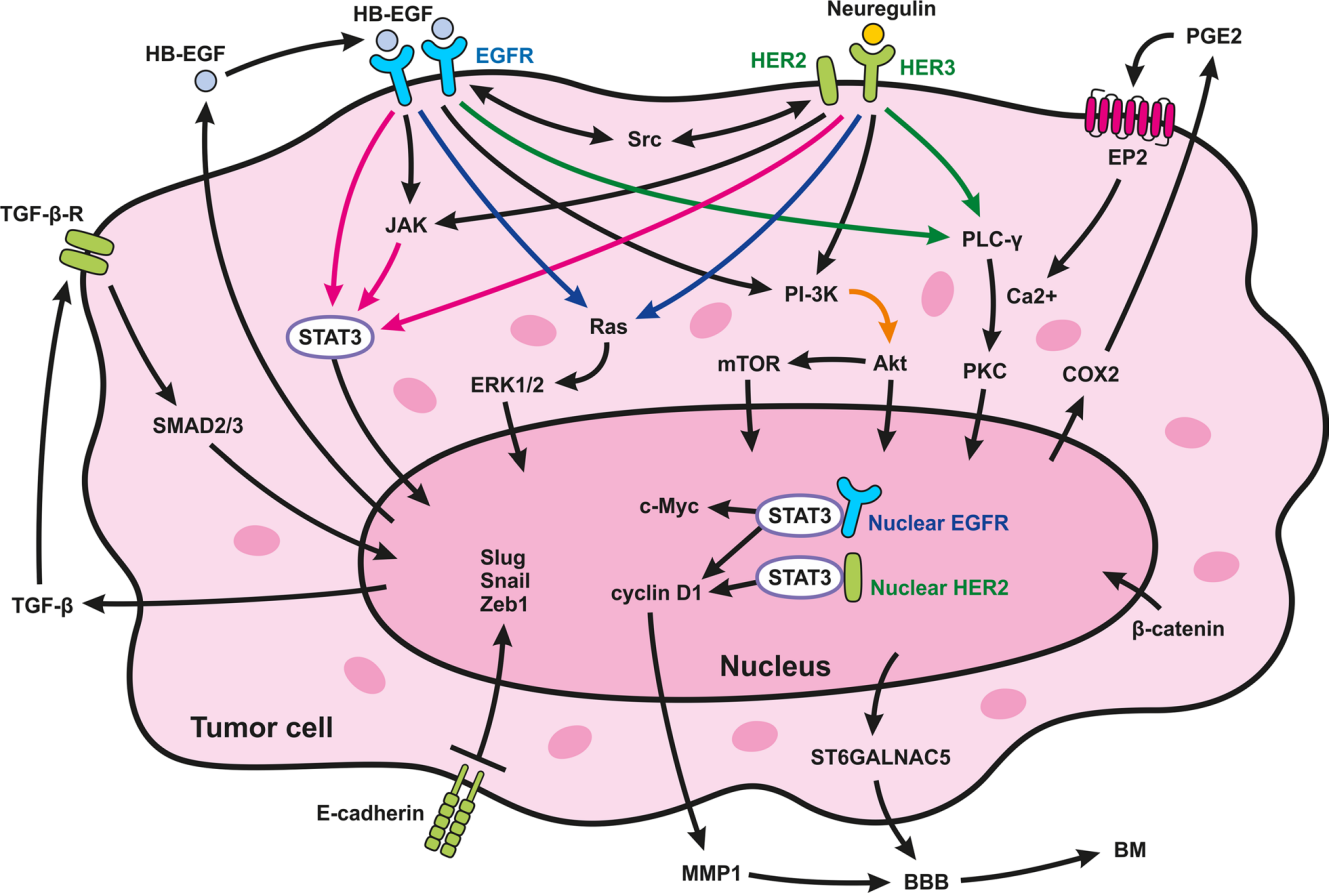
Key signaling pathways involved in breast cancer, lung cancer and melanoma Lung cancer, breast cancer, and melanoma are malignancies that often metastasize to the brain, utilizing specific signaling pathways. In lung cancer, the RAS-RAF-MEK-MAPK pathway (marked by the blue arrow) drives protein activity changes and changes in gene expression, while the inhibition of apoptosis and promotion of cell survival depend on the Akt pathway (marked by the orange arrow). Additionally, activation of PLC-γ results in the synthesis of second messengers, such as inositol 1,4,5-triphosphate (IP3) and 1,2-diacylglycerol (DAG), which are crucial for intracellular Ca2 + release (marked by the green arrow). In breast cancer, HER2-positive subtypes often involve ligand binding to HER3, leading to dimerization with HER2 and initiating phosphorylation events similar to those of EGFR, which drive tumor cell proliferation and brain metastasis. In melanoma, activation of EGFR causes phosphorylation of STAT3 (marked by the pink arrow), followed by its dimerization and transport to the nucleus, promoting gene expression that facilitates brain metastasis. Together, these signaling pathways enhance tumor cell proliferation, survival, and invasive potential, ultimately leading to brain metastasis. c-Myc: cellular Myelocytomatosis; COX2: Cyclooxygenase-2; ERK1/2 (Extracellular Signal-Regulated Kinases 1 and 2); HER: Human epidermal growth factor receptor

HER2, a 185 kDa transmembrane protein encoded by HER2/neu, is closely related to EGFR, although it does not bind to identical ligands. When activated, both hetero and homodimers of HER2 initiate phosphorylation events similar to those of EGFR, activating several signaling pathways implicated in breast cancer progression. These pathways include the STAT3, RAS-MAPK, and PI3K pathways, resulting in the inactivation of proteins that trigger apoptosis and the upregulation of genes that promote cell growth (Fig. 3). HER2 mutations have been identified in lung cancer [138, 139]. Approximately 1–4% of NSCLC cases exhibit HER2 mutations [140], and the expression of a HER2 mutant containing a 12 bp insertion at exon 20 (G776YVMA) was found to be more potent in BM than in wild-type HER2. This mutation is linked to activated signal transducers, EGFR phosphorylation, and the promotion of survival, invasiveness, and tumorigenicity [138, 139]. Recent studies have indicated that the high incidence of BM in patients with primary NSCLC is associated with the G776YVMA insertion [141, 142].

#### Cytokines

Cytokines play pivotal roles in the intricate processes by which metastatic lung cancer cells colonize the brain parenchyma. The release of cytokines, chemokines, and tumor-secreted exosomes primes the brain microenvironment, fostering the growth of lung cancer cells within the brain [143]. Several critical factors and interactions significantly contribute to this complex process. Transforming growth factor-beta 1 (TGF-β1) is widely acknowledged for its role in the promotion of EMT. Khan et al. demonstrated that compared with their wild-type counterparts, lung cancer cells pretreated with TGF-β1 in mouse models presented a nearly threefold increase in their ability to metastasize to the brain [144]. TGF-β1 single nucleotide polymorphisms (SNPs), particularly rs1982073, have been associated with reduced BM-free survival in patients with NSCLC who have undergone radiation therapy. This genetic variant appears to serve as a predictive factor for an elevated risk of BM development in the patient population following radiation treatment [145]. Genotype variants within the TGF-β signaling pathway can also act as predictive biomarkers for BM development in NSCLC patients, offering potential insights into disease progression and treatment response [143].

Furthermore, tumor necrosis factor-alpha (TNF-α) is inversely correlated with N-acetyl-aspartate, an indicator of mitochondrial oxidative capacity in the occipital cortex. Even before BM formation, the cerebral metabolic status of patients with lung cancer is altered, characterized by lower levels of glutamate, creatine, and phosphocreatine in the cortex [146].

In NSCLC samples derived from patients with BMs, there was notable upregulation of the expression of the chemokine CXCL12 and its corresponding receptor CXCR4. CXCR4 plays a pivotal role in communication between cancer cells and their microenvironment [147]. In a study by Chen et al. involving 32 patients with BM originating from NSCLC, the expression patterns of CXCR4 were significantly elevated in 90% of primary tumors and 100% of BMs compared with those in NSCLC patients without distant metastases [148]. Similarly, Paratore et al. reported concordant results, indicating that the immunoreactivity of CXCL12 and CXCR4 was significantly greater in NSCLC samples from patients with BMs. Receiver operating characteristic (ROC) analysis was conducted to determine the optimal cut-off values for CXCL12 and CXCR4 immunoreactivity, enabling discrimination between NSCLC patients with and without BMs. This expression profile demonstrated good diagnostic accuracy and adequate predictive power [149]. Furthermore, the downregulation of C-X3-C motif chemokine receptor 1 (CX3CR1) in lung adenocarcinomas is associated with an increased likelihood of metastatic spread to the brain [150].

Another study demonstrated that the frequency of programmed death-ligand 1 (PD-L1) in myeloid cells is correlated with the presence of BM. Compared with controls, patients with lung carcinoma with BMs presented elevated PD-L1 levels in peripheral monocytes [151]. It has also been revealed that tumor-derived IL-6 can induce PD-L1 expression in myeloid cells. Treating lung cancer cells with brain metastasis-conditioned media with anti-IL-6 or anti-IL-6 receptor antibodies reduced PD-L1 expression in patient-derived BM [152].

#### Other molecules involved in BM

Various other molecules are closely associated with BM formation in primary lung tumors (Table 1). A long non-coding RNA called MALAT1 (metastasis-associated lung adenocarcinoma transcript 1) promotes EMT and BM formation [153]. Not surprisingly, the presence of MALAT1 in primary NSCLC is associated with BM and a poor prognosis [154, 155]. Silencing MALAT1 in invasive lung cancer cells prevents BM formation in mice [156]. In contrast, MALAT1 can have opposing effects on breast cancer, acting as an antimetastatic factor in some cases [157]. In addition, the cell adhesion molecule N-cadherin is overexpressed in the BM of patients with NSCLC [158]. It is a mesenchymal marker belonging to the calcium-dependent adhesion molecule family, which directly mediates cell–cell adhesion and is crucial for cancer progression [159]. In parallel, loss of E-cadherin expression in primary tumors of patients with NSCLC was significantly associated with BM [160]. Moreover, silencing CADM2 in several NSCLC cell lines results in reduced vimentin levels, decreased cell migratory ability, and increased expression of the epithelial marker E-cadherin [161]. Therefore, CADM2 induces EMT and supports the migration of lung cancer cells into the CNS [6]. Tumor necrosis factor-alpha (TNF-α) amplifies the adhesion between CD15 and E-selectin. Studies suggest CD15 facilitates cancer cell adhesion to the brain endothelium, particularly under TNF-α-induced inflammatory conditions [162]. Samah Jassam and colleagues designed experiments using in vitro models to show that blocking CD15 or CD62E significantly reduces cancer cell adhesion to the brain endothelium, highlighting their importance in the early stages of cancer cell extravasation [162].

**Table 1:** Key molecules are involved in brain metastasis, along with the mechanisms by which these molecules contribute to the penetration of tumor cells into the brain

Molecule	Mechanism	Consequences	Reference
ADAM9	Disruption of TJ proteins and extracellular matrix	Metastatic cell transmigration through endothelial cells	[231]
Annexin A2	Activation of several signaling pathways, including the MAPK, NF-κB, and STAT3 pathways	Leading to higher concentrations of IL-6 and tumor TNF-α, which results in the creation of a premetastatic inflammatory microenvironment in distant sites, mainly in the brain	[73]
Cathepsin S	Proteolytic degradation of adhesion molecule JAM-B	Accelerating the penetration of metastatic cells through the BBB	[211]
CD24 and CEA	Bind to E-selectin on endothelial cells	Promoting tumor cell rolling	[225, 226]
COX-2 overexpression	Produced by metastasis cells	Increasing the expression of MMPs elevates the self-renewal capacity of the tumor cells in the brain	[124, 246]
CX3CL1 and CXCL13	Compromising BBB integrity	Attracting metastatic cells	[251]
E-cadherin low expression	Involving in endothelial-mesenchymal transition	Promoting tumor cells local invasion and intravasation	[6, 7]
EGFR	Contributing to several complex signaling cascades, including phosphorylation of STAT3	Causes metastasis (growth, signaling, differentiation, adhesion, migration, and survival of cancer cells)	[245]
ErbB2 activation	Overexpressing MMPs, the attraction of CXCL12, a specific ligand for CXCR4 expressed on brain endothelial cells	Invasiveness of metastatic cells, Migration, and invasion of malignant cells	[138]
E-selectin overexpression on the surface of the ECs or soluble E-selectin	Interacting with CD44 and MUCI that are expressed on the CRCs	Facilitating the shear-resistance adhesion process	[191, 192]
HB-EGF	Activating RAS-RAF-MEK-MAPK, PI-3 K-AKT-mTOR, PLC-γ-Ca2 + /PKC, and JAK-STAT3	Resulting in increased MMP-1, MMP-2, and heparinase, therefore decreased expression of TJ proteins claudin-5 and VE-cadherin	[124]
HCELL	Binding to E-selectin on endothelial cells	Promoting tumor cell rolling	[225, 226]
ICAM-1	Binding to tumor cells	Facilitating metastatic cell rolling and adhesion to endothelial cells	[192]
IL-1β, TNF-α, IFN-γ, CCL2	Playing major roles in inflammatory reactions	Promoting metastatic cell transmigration through endothelial cells	[206, 227, 244]
IL-23 in astrocytes	Upregulation caused by metastatic cells and BBB distribution	Resulting in the induction of secretion of MMP-2	[215]
Integrins	Adhesion to the endothelium as ligands for VCAM-1 and fibronectin, Transmigration, Disruption of TJ proteins and extracellular matrix	Adhesion of metastatic cells	[224, 225]
KRAS	Functioning as a transducer of signals that activate growth factor receptors	Promoting lung cancer-associated brain metastasis	[132–135]
LAMP1/2	Adhesion- ligand for galectin-3	Increasing tumor adhesion to endothelial cells	[225]
miR-181c	Downregulates the 3phosphoinositide-dependent protein kinase-1 (PDPK1)	Causing TJ alteration and BBB integrity	[72]
MMPs	Disruption of TJ proteins and extracellular matrix	Accelerating the penetration of metastatic cells through the BBB	[69, 165, 211]
MUC1	Involving in shear-resistant adhesion	Promoting metastatic cell rolling and adhesion to endothelial cells	[184]
N-cadherin overexpression	Involving in endothelial-mesenchymal transition	Facilitating tumor cells local invasion and intravasation	[6, 7]
Neuregulin-1 overexpression on endothelial cells	Causing heterodimerization of HER2 with HER3	Resulting in increased expression of MMPs such as MMP-9 and adhesive molecule ICAM-1	[252]
NGF	Chemoattractant to ADAM9 overexpressed in brain metastatic cells	Causing penetration of metastatic cells, Survival of malignant cells in the CNS	[243, 244]
PCDH7 on cancer cells	Interacting with Cx43 on astrocytes	Ultimately, it promotes by activating the STAT1 and NF-kB signaling pathways	[182, 183]
Plasmin	Produced by activation of uPA and tPA	Disrupting the TJ proteins and extracellular matrix	[233]
PLEKHA5	Downstream product of Met signaling	Promoting migration of metastatic cells and their integration into host tissue	[253]
PSGL-1	Binding to E-selectin on endothelial cells	Facilitating tumor cell rolling	[225, 226]
pSTAT3 + reactive astrocytes	Upregulating certain immunosuppressive molecules such as VEGF-α	Preventing the access of CD8 + cytotoxic T cells to cancer cells	[106, 177]
Rho/ROCK activation	Involving in TJ reassembly	Transmigration of cancer cells	[287]
S100 protein and lipocortin precursor	Produced by activated astrocytes	Supporting the survival of metastatic cells in the CNS	[243]
Seprase in metastatic cells	Pericellular proteolysis, Disruption of TJ protein, and extracellular matrix	ECM degradation (including collagen I and III) and invadopodia	[236, 237]
Serine proteases	Disruption of TJ proteins and extracellular matrix	Accelerating the penetration of metastatic cells through the BBB	[220]
SIRT1	Leads to higher secretion of exosomes	Promoting migration of cancer cells	[87]
ST6GALNAC5	Catalyzing α-series gangliosides, GD1α	Involving in the transmigration of metastatic cells through the brain endothelium	[241, 242]
Thomsen-Friedenreich antigen (TA)- CD44v6	Adhesion- ligand for galectin-3	Increasing tumor adhesion to endothelial cells	[224]
VEGF	Rearranging the cytoskeletal and decreasing integrity of the endothelial layer	Promoting tumor cell invasion	[161, 213]
Xist downregulation	Induction of epithelial-mesenchymal transition	Results in the secretion of exosomal miR-503, which promotes the transition of tumor-suppressive (M1) to tumor-promoting (M2) phenotype of microglia	[185, 186]

Additional factors, such as cystatin C, cathepsin L, insulin-like growth factor-binding protein 7 (IGFBP7), and vascular endothelial growth factor (VEGF), are secreted by NSCLC cells that metastasize to the brain [163]. These factors contribute to the impairment of the endothelial glycocalyx, resulting in the upregulation of E-selectin and facilitating the adhesion of metastatic cells to the microvascular endothelium of the brain [143].

CD44, a non-kinase cell surface glycoprotein, constitutes a substantial family of cell adhesion molecules (CAMs) that exhibit diverse functions, such as interactions with ECM components, cytokines, and growth factors secreted by cells within the tumor microenvironment [164]. The entire CD44 family is encoded by a single gene comprising 19 exons [165, 166]. CD44 is present in embryonic stem cells, connective tissue, and bone marrow and has been recognized as a marker for cancer stem cells across a spectrum of tumors [166]. In this respect, a study on 15 patients with BM originating from primary NSCLC tumors demonstrated that a deficiency in CD44 protein expression is associated with increased metastatic potential of NSCLCs [164].

miRs are crucial regulators of BBB permeability, influencing tumor cell extravasation into the brain microenvironment. For instance, in NSCLC, Wu et al. demonstrated that miR-1207-5p targets Erythrocyte Membrane Protein Band 4.1 Like 5 (EPB41L5) [167]. EPB41L5, a member of the band 4.1 protein family, is essential for maintaining the structural integrity and function of the cell membrane [168]. It promotes EMT, disrupts TJs, and increases BBB permeability. By targeting EPB41L5, miR-1207-5p exerts a tumor-suppressive role, helping to maintain BBB integrity and limit metastatic spread to the brain [167, 169]. Similarly, Li et al. found that miR-596-3p targets Yes-associated protein 1 (YAP1) in NSCLC. YAP1 is pivotal in cellular responses to growth signals, and its dysregulation has been linked to various cancers [170]. YAP1 promotes MMP-2 activity, which degrades TJ and impacts BBB permeability [171].

In BM from breast cancer, it was shown that miR-509 targets TNFα and RhoC. RhoC is a member of the Rho family of small GTPases, which are critical regulators of various cellular processes, including cytoskeletal dynamics, cell migration, cell cycle progression, and gene expression [172]. This targeting effectively inhibits RhoC-induced MMP-9 activity, which also disrupts BBB TJ [171].

### Metastasis of breast cancer to brain tissue

Breast cancer (BC) is the most common cancer in the female population [173], with 2.26 million diagnosed cases and 685 thousand reported deaths worldwide in 2020. The clinical categorization of BC depends on the expression of estrogen receptor (ER), progesterone receptor (PR), and HER2/Neu, as well as on the value of Ki-67 [174, 175].

HER2 is significantly overexpressed in invasive BC, ranging from 15–35% [176]. In addition to its role in breast cancer, HER2 overexpression has also been observed in other types of cancer [177]. The functional tyrosine kinase domain of HER2 can be activated via interactions with ligand-activated EGFR or HER3. Like EGFR, activated HER2, whether hetero or homodimers, initiates phosphorylation events, activating several signaling pathways implicated in BC progression [176]. These pathways, including the STAT3, RAS-MAPK, and PI3K pathways, result in the inactivation of proteins that trigger apoptosis and the upregulation of genes that promote cell growth, thereby facilitating tumor cell proliferation [176]. Recent research has revealed that HER2 directly interacts with the proapoptotic protein p53 upregulated modulator of apoptosis (PUMA) and phosphorylates PUMA, leading to its degradation and promotion of tumor cell survival [178]. As described below, numerous other factors are involved in BC metastasis to the brain.

#### STAT3 pathway

The distinctive brain microenvironment inadvertently facilitates the development and expansion of the BM. A subset of reactive astrocytes (RA), activated by STAT3, surrounds BMs [179]. STAT3, a member of the STAT family, plays a role in cell growth, apoptosis, tumor progression, and differentiation [180]. Tumor cells release various cytokines that induce reactivity in astrocytes. The interaction between cytokines and growth factors with their respective receptors activates a critical signaling pathway, the Janus kinase (JAK)-signal transducer and activator of transcription (STAT) pathway [181]. These STAT3 + reactive astrocytes generate cytokines and other substances that impede the body's appropriate response to the tumor, thereby compromising both the innate and adaptive immune systems [179].

Recent investigations have revealed that a significant proportion of astrocytes identified in BMs exhibit phosphorylated (active) STAT3 (pSTAT3), indicating the importance of STAT3 signaling in tumor-associated cells [182]. pSTAT3 + reactive astrocytes hinder the access of CD8 + cytotoxic T cells to cancer cells by increasing the expression of specific immunosuppressive molecules, such as programmed cell death-1 ligand 1 (PD-L1), VEGF-A, lipocalin-2, and tissue inhibitor of metalloproteinases-1 (TIMP-1). In primary brain tumors, tumor-associated astrocytes express STAT3 and PD-L1 at elevated levels, contributing to an immunosuppressive environment by producing additional cytokines such as IL-10 and TGF-b [180, 10972].

RA can also shield cancer cells from chemotherapy by upregulating the expression of specific genes, which is essential for generating gap junctions between the two cell types [183]. The interaction between protocadherin 7 (PCDH7) on cancer cells and Cx43 on astrocytes facilitates the creation of these gap junctions. Through these gap junctions, cancer cells stimulate the cGAS-STING (stimulator of interferon genes) pathway in astrocytes via 2′,3′-cyclic GMP-AMP (cGAMP), increasing the production of potent inflammatory cytokines, such as interferon-α (IFN-α) and tumor necrosis factor [184, 185]. These cytokines can promote BM in cancer cells by activating the STAT1 and nuclear factor kappa-light-chain-enhancer of activated B cells (NF-kB) signaling pathways [108].

Moreover, nuclear EGFR, in collaboration with STAT3, has been demonstrated to directly upregulate genes that facilitate the BM process [177]. EGFR activation has additional effects, including direct phosphorylation of the STAT3 transcription factor and its translocation to the nucleus for the regulation of gene expression. EGFR can translocate to the nucleus, influence gene expression, phosphorylate nuclear proteins, and alter their functions. Increased nuclear EGFR expression has been associated with poor clinical outcomes in various cancers. Additionally, EGFR can translocate to the mitochondria to counteract therapy-induced apoptosis [177] (Fig. 3).

BC most frequently metastasizes to three distinct anatomical regions in the brain. The brain parenchyma is the most common site, with 78% of cases presenting with multiple metastases and 14% presenting with solitary metastases. Additionally, 8% of metastases occur in the leptomeninges, and the choroid plexus is considered a sanctuary site for breast cancer metastasis [186].

Xist is a 19 kb long non-coding RNA that originates from the inactive X chromosome and plays a crucial role in X chromosome inactivation in female cells [187]. A study by Xing et al. demonstrated significant downregulation of Xist in metastatic brain tissue, leading to the induction of EMT through c-Met activation [74, 188]. c-Met is a tyrosine kinase receptor that binds to hepatocyte growth factor and subsequently activates various cellular signaling pathways crucial for proliferation, motility, migration, and invasion. Mutations in c-Met are associated with various human cancers [189]. Furthermore, the loss of Xist results in the secretion of exosomal microRNA-503, promoting the transition of microglia, a key component of the brain's innate immune system, from a tumor-suppressive (M1 pro-inflammatory) phenotype to a tumor-promoting (M2 anti-inflammatory) phenotype [74]. This transition allows metastatic tumor cells to overcome the cytotoxic effects of M1 microglia and facilitate their growth.

Microglia pose a significant challenge to overcome, as they are integral to the innate immune system and release a substantial number of cytokines and inflammatory molecules upon activation by inflammation or cancer cells [188]. The primary cytokines associated with the M1 phenotype include interferon-gamma (IFN-γ), tumor necrosis factor-alpha (TNF-α), IL-2, and IL-12. In contrast, the M2 phenotype is correlated with cytokines such as IL-4, IL-10, IL-13, and TGF-β [190]. Metastatic tumor cells employ two strategies to evade the immune defense mechanism: first, by avoiding the cytotoxic effects of the M1 phenotype, and second, by inducing conversion from the M1 phenotype to the M2 phenotype [188].

Huang et al. investigated the correlation between the PI3K/AKT cellular pathway and Xist, revealing an inverse relationship. Consequently, as the level of Xist decreases in cells, the level of pAKT increases [74]. Activation of the PI3K pathway has been observed in 70% of BC patients with BM [191]. Moreover, downregulation of Xist has been detected in 78% of primary tumors in patients with BC-derived BMs [188].

#### Cell adhesion molecular markers

As circulating tumor cells (CTCs) traverse the bloodstream, they eventually become trapped in microvessels within the brain, particularly at the branching points of the vasculature, initiating adhesion to endothelial cells (ECs). The interaction between E-selectin on the EC surface and CD44 and MUC1 facilitates shear-resistant adhesion, particularly at branching sections of the vasculature [186]. The expression of E-selectin is provisional and can be released into the circulation in a soluble form, or E-selectin may be internalized after activation. Notably, there have been reports of high levels of soluble E-selectin in the serum of patients with metastatic breast cancer [192]. The overexpression and abnormal glycosylation of MUC1 are commonly observed in various cancers. Physiologically, MUC1 is 5–10 times larger than most membrane proteins because its repeating units are 20 identical amino acid sequences rich in serine and threonine. Abnormal MUC1 with shorter oligosaccharides can efficiently bind E-selectin [193].

Brain metastasis is observed in 10–30% of women diagnosed with stage IV breast cancer [108], with the highest occurrence in the triple-negative subtype (25–27%), followed by the HER2 + subtype (11–20%) [175]. The risk of BM is notably higher in patients with HER2-positive breast cancer or triple-negative breast cancer (TNBC) than in those with luminal BC. The estimated frequency of BMs ranges from 20–30% in patients with HER2-positive breast cancer and TNBC, whereas it is less than 10% in patients with the luminal subtype [108].HER2 promotes the phosphorylation of proteins across multiple signaling pathways, suppresses pro-apoptotic proteins, and enhances the expression of genes related to cell proliferation, processes that contribute to epithelial-mesenchymal transition (EMT) and increase the risk of brain metastasis [194–196]. Notably, in aggressive breast cancers, HER2 is often co-expressed with junctional adhesion molecule-A (JAM-A), a cell–cell adhesion protein involved in tight junction formation in epithelial and endothelial cells, further supporting tumor progression and metastatic [197, 198].. HER2-overexpressing BC cells induce the expression of transcription factors such as Snail, Slug, and ZEB1, leading to increased TGF-β production, elevated N-cadherin levels, and reduced E-cadherin and cytokeratin-18 expression [38]. Targeting HER2 has been suggested as a potential therapeutic strategy to inhibit metastasis, as HER2 inhibition has been shown to reduce metastasis not only to the brain but also to the lungs and liver in mouse models.

Another regulator of E-cadherin is SNORA17B, a small nucleolar RNA (snoRNA) involved in ribosomal RNA methylation. SNORA17B is highly expressed in the brain metastases of patients with breast cancer and is associated with a poor prognosis. SNORA17B-transfected human breast cancer cell lines display increased invasive ability and reduced E-cadherin expression, suggesting its involvement in controlling E-cadherin levels [6].

Astrocyte elevated gene-1 (AEG-1) is another molecule that links EMT with BM. AEG-1 overexpression upregulated N-cadherin and reduced E-cadherin and ZO-1 expression. AEG-1 is an endoplasmic reticulum (ER)-associated cytoplasmic RNA-binding protein that interacts with numerous mRNAs encoding secretory, cytosolic, and organelle proteins [199]. Initially discovered in primary astrocytes of the human fetus and mainly localized in the ER, AEG-1 was later found in the cell membranes of breast cancer cells. Its roles in cancer include promoting malignant transformation, resistance to chemotherapy, anoikis, angiogenesis, and metastatic spread [41, 42, 200].

In a study conducted by Wu et al., the roles of MMP1 and cyclooxygenase 2 (COX2) in breast cancer brain metastasis were investigated [201]. This study demonstrated increased expression of MMP1 and COX2 in brain metastatic cancer cells. MMP1 plays a role in degrading claudin and occludin [201], which are crucial transmembrane proteins integral to tight junctions [202]. This study also revealed the upregulation of COX2 in brain metastatic cells. COX2 induces the secretion of prostaglandins, which in turn enhances the expression of MMP1. Moreover, COX2 and prostaglandins can stimulate astrocytes to release CCL7, thereby increasing the self-renewal capacity of tumor cells in the brain. In support of this, when MMP1 and COX2 are knocked down in brain metastatic cells, their ability to metastasize to the brain is reduced [32].

### Metastasis of melanoma to brain tissue

Melanoma, a malignancy originating from melanocytes responsible for melanin production and derived from the neural crest [203], presents a notable complication in advanced stages, with BMs affecting nearly half of the patients and contributing up to 54% of melanoma-related mortalities [204]. Among these BMs, approximately 49% are intraparenchymal, 22% are leptomeningeal, and 32% are dural [205, 206].

The development of melanoma BMs (MBMs) is preceded by early changes in the brain microenvironment, including breakdown of the BBB, increased vascular permeability, and reactive astrogliosis. Schwartz et al. revealed the upregulation of pro-inflammatory cytokines such as CXCL10, CCL17, CCL2, IL-6, and IL-1β during MBM [96].

Studies indicate that CXCL10 influences the migration of monocytes, macrophages, T cells, and natural killer (NK) cells to the brain [207]. Patients with advanced melanoma, which is associated with a poor prognosis, have higher levels of CXCL10 [208, 209]. Furthermore, the receptors for CXCL10 and CXCR3 are upregulated in melanoma cells, with a predilection for the brain [210]. Immunokine profiling studies in the CSF of patients with advanced melanoma suggested that increased levels of CXCL10, CCL17, and CCL4 may be correlated with a more aggressive development of BMs [211].

MMP-2 has emerged as a crucial factor, exhibiting heightened expression correlating with increased malignancy, particularly in the most metastatic cell lines, MV3 and BLM. The primary focus is on the mRNA and protein expression patterns of MMP-1, MMP-2, MMP-3, MMP-9, TIMP-1, and TIMP-2, both in vitro and in vivo. Additionally, they revealed that MMP-1, although detectable in all cell lines at the mRNA level, has limited protein expression [212].

In terms of STAT3, MBM cells exhibit increased activity of STAT3, AKT [143, 178, 213], and AKT. STAT3 activity in the MBM triggers a cascade of events that contribute to the potential of the BM, including angiogenesis, cell invasion, MMP-2 secretion, cytokine expression, and immune suppression [178]. In melanoma cell lines, the loss of suppressor of cytokine signaling-1 (SOCS-1) expression is correlated with increased STAT3 signaling and subsequent overexpression of MMP-2, basic fibroblast growth factor (bFGF), and VEGF [214]. This, in turn, increases invasion and angiogenesis in melanoma cells, facilitating the development of MBM [214].

Patel et al. suggested that STAT3 activation is a key contributor to the occurrence of melanoma-derived BMs, as evidenced in a study of 216 autopsied metastatic melanoma specimens [215]. Moreover, brain-metastasizing melanoma cells have the capacity to reprogram astrocytes, leading to the expression of the pro-inflammatory cytokine IL-23, which in turn increases MMP-2 levels and facilitates melanoma cell migration and invasion into the brain parenchyma. Conversely, reduced MMP-2 expression in melanoma cells has been linked to the inhibition of IL-23-induced invasiveness [216].

## Brain barriers in relation to brain metastases and metastatic spread

Most BMs are found in the brain parenchyma, dura, and leptomeninges [217, 218] and, to a lesser degree, in the pituitary and pineal glands [217]. One of the main characteristics of primary and brain metastases is the formation of surrounding vasogenic brain edema, which reflects damage to the BBB [219, 220]. The relative frequency of metastases in different brain regions can be influenced by several factors, including brain anatomy and the type of primary tumor.

Generally, metastatic cells can reach brain tissue through the paracellular pathway or through transcellular migration. However, low transcytosis, together with the limited permeability of TJs, makes it challenging for metastatic cells to cross the BBB and reach brain tissue. The paracellular pathway is characterized by the alteration of TJ proteins and the apoptosis of endothelial cells [221, 222]. The extravasation of metastatic cells shares similarities with that of leukocytes, revealing three main steps in the extravasation process: rolling, adhesion, and transmigration (diapedesis).

Brain metastases are most commonly located in the supratentorial region of the brain above the tentorium cerebelli in the cortex and white matter of the brain. In this area, blood flow is slower, allowing metastatic cells to adhere to the capillary wall.

In contrast, the hippocampal region is a preserved part of the brain with a relatively lower incidence of BMs, probably due to a different vessel supply (via the posterior cerebral artery) or due to differences in the BBB related to the unique presence of neural stem cells involved in new memory formation [223]. During whole-brain radiotherapy, the hippocampus is avoided to preserve neurocognitive functions, including memory, due to the relatively low risk of the presence of BM [224].

### Blood–brain barrier in relation to brain metastases

#### Metastatic cells rolling and adhesion to endothelial cells

**Fig. 4:**
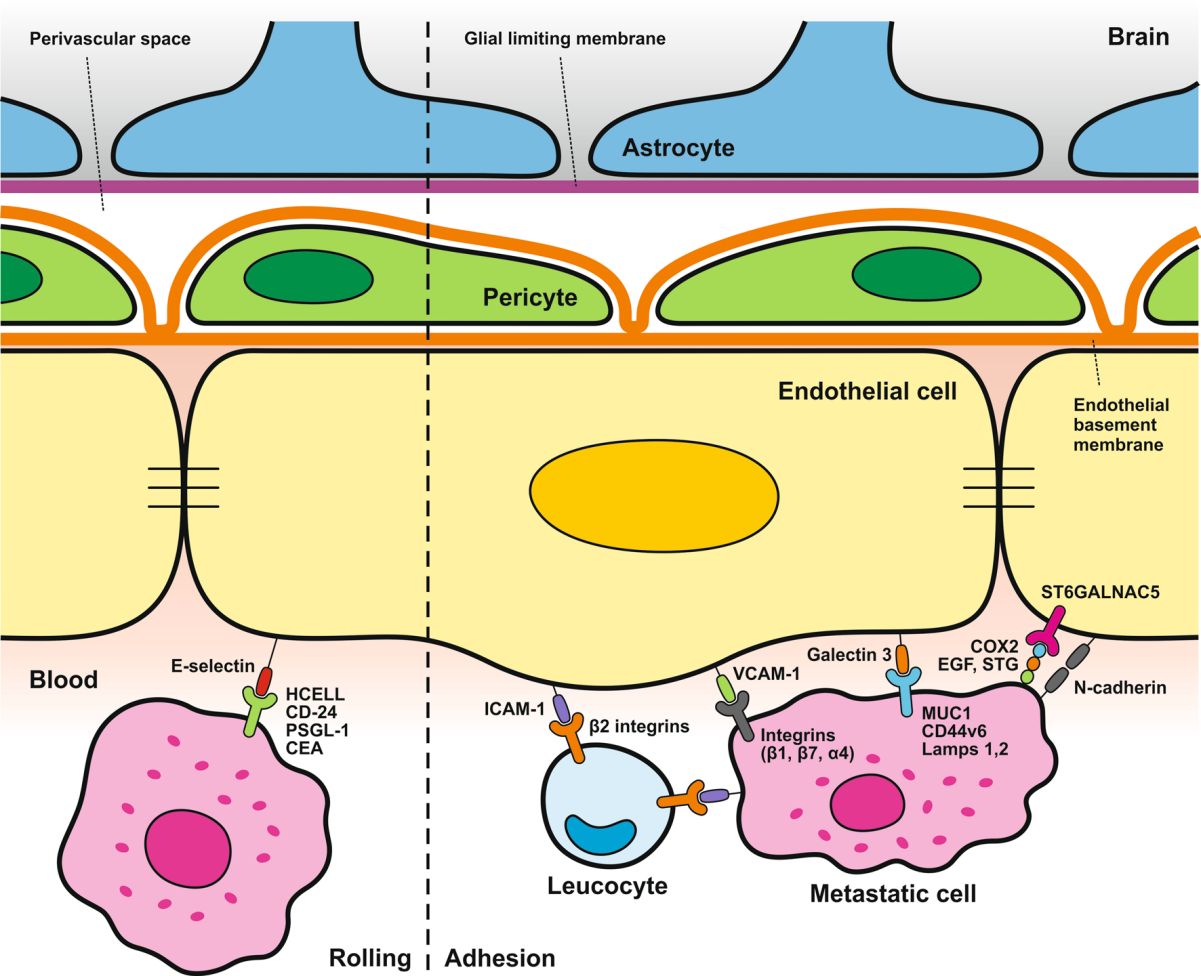
Mechanisms of Metastatic Cell Rolling and Adhesion to Brain Endothelial Cells. This schematic illustrates the endothelial cell layer, pericytes, and astrocytes forming the blood–brain barrier, with metastatic cells depicted in the process of adhering to endothelial cells facilitated by various molecular interactions. **Rolling phase**: Endothelial cells express E-selectin, which binds to glycoproteins such as HCELL, CD24, PSGL-1, and CEA on the surface of metastatic cells, initiating the rolling process. **The adhesion phase** includes the following steps: (**1)** Leukocyte bridging: Metastatic cells use leukocytes (which express β2 integrins) as intermediaries to bind to ICAM-1 on endothelial cells. (**2)** Direct adhesion: Metastatic cells also express integrins (α4, β1, β7) that interact with VCAM-1 on endothelial cells, facilitating direct adhesion. Additionally, MUC1, CD44v6, and lysosomal-associated membrane proteins (Lamps) 1 and 2 on metastatic cells bind to galectin-3 on endothelial cells, enhancing adhesion. **3)** N-cadherin interaction: N-cadherin on metastatic cells interacts with N-cadherin on brain endothelial cells, promoting stable cell contact

The rolling step is probably the initial phase of the adhesion of metastatic cells to the brain endothelium, similar to immune cells. The key molecules mediating tumor cell rolling and adhesion are glycoproteins, integrins, galectins, and the Thomsen-Friedenreich antigen, glycoproteins present on the surface of most human cancer cell types [225, 226]. These molecules serve as ligands for the adhesion receptors expressed on the surface of endothelial cells. During the rolling step, E-selectin on the surface of endothelial cells recognizes and binds to glycoproteins, such as HCELL, PSGL-1, CD24, and CEA, which are ligands that bind to L-selectin (Fig. 4). N-cadherin, a member of the calcium-dependent adhesion molecule family localized on the brain endothelium, mediates cell contact via its expression on the metastatic cell surface. Metastatic cells can use leukocytes as bridging cells to promote adhesion through ICAM-1, an adhesion molecule present on the surface of endothelial and tumor cells (Fig. 4). Because metastatic cells, unlike leukocytes, do not express β2-integrins, ligands required to interact with ICAM-1, the adhesion of tumor cells occurs by bridging by immune cells [226, 227]. The mechanisms of adhesion of metastatic cells to the endothelium include the expression of α4, β1, and β7 integrins, which act as ligands for vascular cell adhesion protein 1 (VCAM-1) and fibronectin. As mentioned above, the Thomsen-Friedenreich antigen, represented by MUC1 and CD44v6, and lysosomal membrane-associated glycoproteins (Lamps) 1 and 2 serve as ligands for galectin-3, thus increasing tumor adhesion to endothelial cells [226].

#### Metastatic cell transmigration through endothelial cells

##### Role of proteolytic enzymes

Metastatic cells squeeze among endothelial cells and disrupt the main TJ transmembrane proteins, such as occludin, claudin-5, and the cytoplasmic protein ZO-1 [228]. The disruption of TJ proteins within endothelial cells and the ECM depends on the accumulation of proteolytic enzymes. The ECM and basement membrane are composed of many different components; therefore, their disintegration, subsequent migration, and invasion of metastatic cells require enzymes with distinct substrate specificities. Increased expression of proteolytic enzymes and other harmful molecules results from specific interactions between metastatic cells and the cellular components of the BBB.

**Fig. 5:**
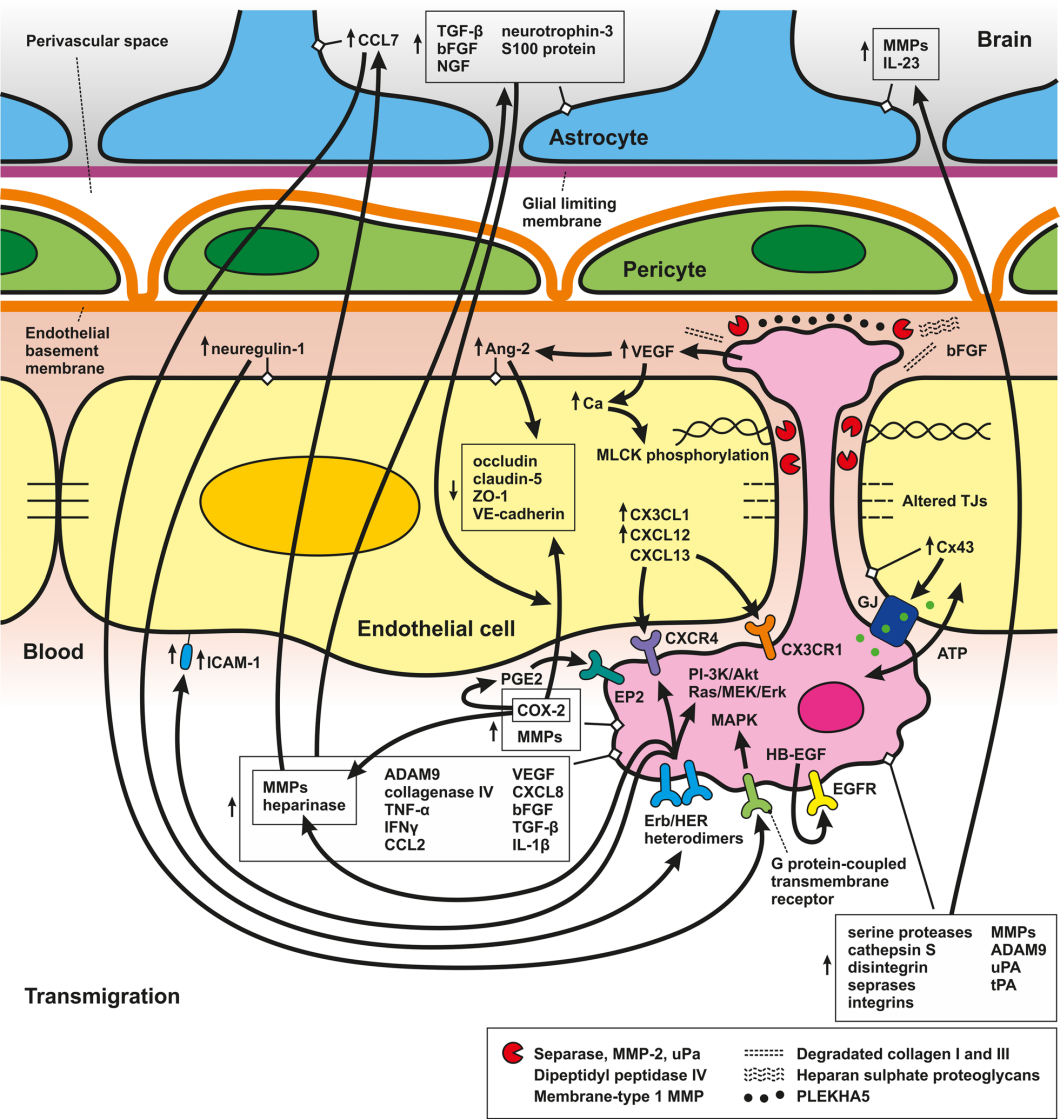
Mechanisms of Metastatic Cell Transmigration through Brain Endothelial Cells. This figure represents the transmigration of metastatic cells across the BBB, which is a complex process involving all components of the BBB. Interaction between metastatic cells and BBB components results in an increase in pro-inflammatory cytokines, chemokines, receptors, proteases, and growth factors. These molecules create a suitable microenvironment for metastatic cells to survive and spread. Several proteolytic enzymes, including separase, MMP-2, uPa, dipeptidyl peptidase IV, and membrane-type 1 MMP causing ECM degradation, are highly expressed on the surface of metastatic protrusions called invadopodia. Communication between metastatic and endothelial via the gap junctions contributes to metastatic cell spread, survival and growth

These “harmful” molecules encompass various proteolytic enzymes and signaling molecules that metastatic cells use to breach the BBB. They compromise BBB integrity and can damage surrounding brain tissue, promoting invasion and growth of metastatic cells within the brain. In the following section, we will discuss these molecules in more detail. The enzymatic system triggered by the interaction of these cells catalyzes the proteolysis of TJ proteins and basement membrane components, such as collagen, laminin, or fibronectin proteoglycans, and activates other enzymes, accelerating the penetration of metastatic cells through the BBB [229]. These enzymes include serine proteases, seprases, MMPs, cathepsin S, disintegrin, metalloproteinase domain-containing protein 9 (ADAM9), urokinase-type plasminogen activator (uPA), and the plasminogen/plasmin system [212, 221, 230–233] (Fig. 5).

The fibrinolytic enzyme plasmin is generated after the activation of plasminogen-by-uPA and tissue-type plasminogen activator (tPA) and is expressed on the surface of metastatic cells [234]. The conversion of plasminogen into plasmin may be enhanced by membrane-bound melanotransferrin, which stimulates the activation of plasminogen through tPA and uPA [235, 236]. Several proteolytic enzymes, such as seprase associated with α3β1 integrin, dipeptidyl peptidase IV, MMP-2, membrane-type 1 MMP, and uPA, are highly expressed on the metastatic cell surface of cell protrusions associated with sites of ECM degradation, called invadopodia [237, 238]. Separases work synchronously with other proteinases, such as MMPs. This synchronized action leads to the cleavage of partially degraded or denatured ECM components, including collagen I and III [238]. In addition, pericellular proteolysis is potentiated by the formation of supramolecular lytic complexes in the plasma membranes of invading metastatic cells. These supramolecular structures are dependent on both the cytoskeleton and integrins, especially β1-integrin [230]. ECM and BM heparan sulfate proteoglycans are considered storage depots for various cytokines and growth factors. The ECM heparan sulfate proteoglycans contain angiogenic factors, such as basic fibroblast growth factor (bFGF), which are released from the ECM heparan sulfate proteoglycans and can support the induction of neovascularization during the migration of tumor cells through the BBB [239]. Moreover, there is evidence that metastatic cells upregulate the expression of some factors, such as IL-23, in astrocytes, which facilitates the progression of BBB disruption by inducing the secretion of MMP-2 [216]. Activated astrocytes play a direct role in tumor metastasis and BBB disruption through the secretion of MMP-2 and MMP-9 [240]. Astrocytes and microglia are immediately localized in the vicinity of metastatic cells and contribute to the progression of cancer cells through the secretion of MMPs [241].

Extravasation through nonfenestrated brain capillaries is influenced by the expression of COX-2, heparin-binding EGF-like growth factor receptor, and ST6 N-acetylgalactosaminide alpha-2,6-sialyltransferase 5 (ST6GALNAC5) in metastatic cells [242]. ST6GALNAC5, a brain-specific sialyltransferase, catalyzes the addition of sialic acid to gangliosides and glycoproteins. Cell-surface sialylation enhances the adhesion and subsequent transmigration of metastatic cells through the brain endothelium [242, 243].

##### Role of trophic factors and neuroinflammation

Activated astrocytes produce and release trophic factors, including neurotrophin-3, TGF-β, or bFGF, as well as nerve growth factor (NGF), S100 protein, and lipocortin precursors to β-melanocyte-stimulating hormone. These molecules support the survival of metastatic cells in the CNS [244]. The penetration of metastatic cells into the neurotrophin-rich stromal brain microenvironment is potentiated by NGF, which serves as a chemoattractant for ADAM9 overexpression in metastatic brain cells [232]. Moreover, in response to NGF, metastatic cells express invasive enzymes such as MMP-2, type IV collagenase, and heparinase [245]. The synthesis of neurotrophins from adjacent brain tissue is also potentiated by cytokines, chemokines, and other inflammatory molecules produced by metastatic cells, including VEGF, bFGF, TGF-β, IL-1β, TNF-α, interferon-gamma (IFNγ), CCL2, C-X-C motif chemokine ligand (CXCL) 8, and COX2, which are major players in inflammatory reactions [228, 245]. Increased expression of COX-2, an inducible pro-inflammatory enzyme, and subsequently MMP-1 was associated with decreased expression of the TJ proteins claudin-5 and VE-cadherin [246].

The expression of invasive enzymes such as MMPs and heparinase is potentiated by autocrine activation of EGFR by HB-EGF and EP2 receptors by PGE2 generated by COX-2 [126, 247]. The pro-inflammatory enzyme COX-2 is involved in the generation of prostaglandins, which are involved in tumorigenesis [201]. These prostaglandins activate astrocytes to generate a niche for metastatic cells by increasing the expression of chemokine (C–C motif) ligand 7 (CCL7), which promotes self-renewal of metastatic cells. CCL7 activates G protein-coupled transmembrane receptors and transmits intracellular signals via the MAPK pathway [212]. The subsequent cytoskeletal rearrangement and decreased integrity of the endothelial layer are partially caused by vascular endothelial growth factor (VEGF). Rearranging the cytoskeleton is promoted by VEGF-induced upregulation of cytosolic calcium levels, leading to the phosphorylation and activation of myosin light chain kinase, resulting in the contraction of endothelial cells [248]. In the case of breast cancer, VEGF also activates endothelial cells that increase the secretion of angiopoietin-2 (Ang-2), one of the molecules involved in the initial step of the metastasis process. Increased secretion of Ang-2 leads to decreased expression of TJ proteins, resulting in alterations in the BBB [249].

The overexpression of MMPs and thus invasiveness, as well as the proliferation of metastatic cells, is potentiated by signaling cascades, including the PI-3 K/Akt and Ras/MEK/Erk cascades. Initiation of these pathways results from the amplification of transmembrane tyrosine kinase receptors from the ErbB family, particularly ErbB2, which forms heterodimers with other members, including ErbB1/HER1, ErbB3/HER3, and ErbB4/HER4 [250]. Moreover, ErbB2 induces the upregulation of chemokine receptor 4 (CXCR4) in metastatic cells. SDF-1α (CXCL12), a specific ligand for CXCR4 expressed in various cells, including brain endothelial cells, contributes to the migration and invasion of malignant cells through the BBB [250, 251]. Another specific ligand belonging to the CXC chemokine family, CX3CL1 and CXCL13, may have barrier-compromising effects by affecting BBB integrity in vitro. These chemokines are part of the endothelial inflammatory phenotype, and high levels of CX3CL1 may attract metastatic cells expressing its receptor CX3CR1 [252]. As mentioned above, the activation of downstream cascades via heterodimerization of the human epidermal growth factor ErbB2/HER2 with ErbB3/HER3 is potentiated by neuregulin-1. Increased expression of neuregulin-1 was found in brain endothelial cells; therefore, neuregulin-1-HER2/ErbB2-HER3/ErbB3 signaling likely promotes metastatic cell transmigration through the BBB by increasing the expression of MMPs such as MMP-9 and the adhesive molecule ICAM-1 [253]. Following extravasation, metastatic cells remain in direct contact with abluminal endothelial cells of the brain capillaries. The location of metastatic cells in the perivascular space in a pericyte-like position provides access to the oxygen and other nutrients necessary for successful proliferation [254]. The subsequent migration of metastatic cells is likely promoted by pleckstrin homology domain-containing A5 (PLEKHA5), a protein that is tyrosine-phosphorylated downstream of Met signaling. PLEKHA5 is associated with microtubules and is expressed at the plasma membrane of the leading edge of motile cells, suggesting its role in cell–cell contact and the migration of metastatic cells [255]. Metastatic cell integration into foreign tissue after they leave circulation and perivascular microtumor formation depends on the creation of more favorable conditions in a foreign microenvironment. Thus, the formation of GJs in the Cx43-rich brain endothelium may play an important role. GJ communication between metastatic and endothelial cells mediates the transfer of ATP, small peptides, ions, and small regulatory RNAs, which are crucial for tumor cell survival and growth [256].

#####  3.2. Blood-arachnoid barrier (leptomeninges) in brain metastasis

**Fig. 6:**
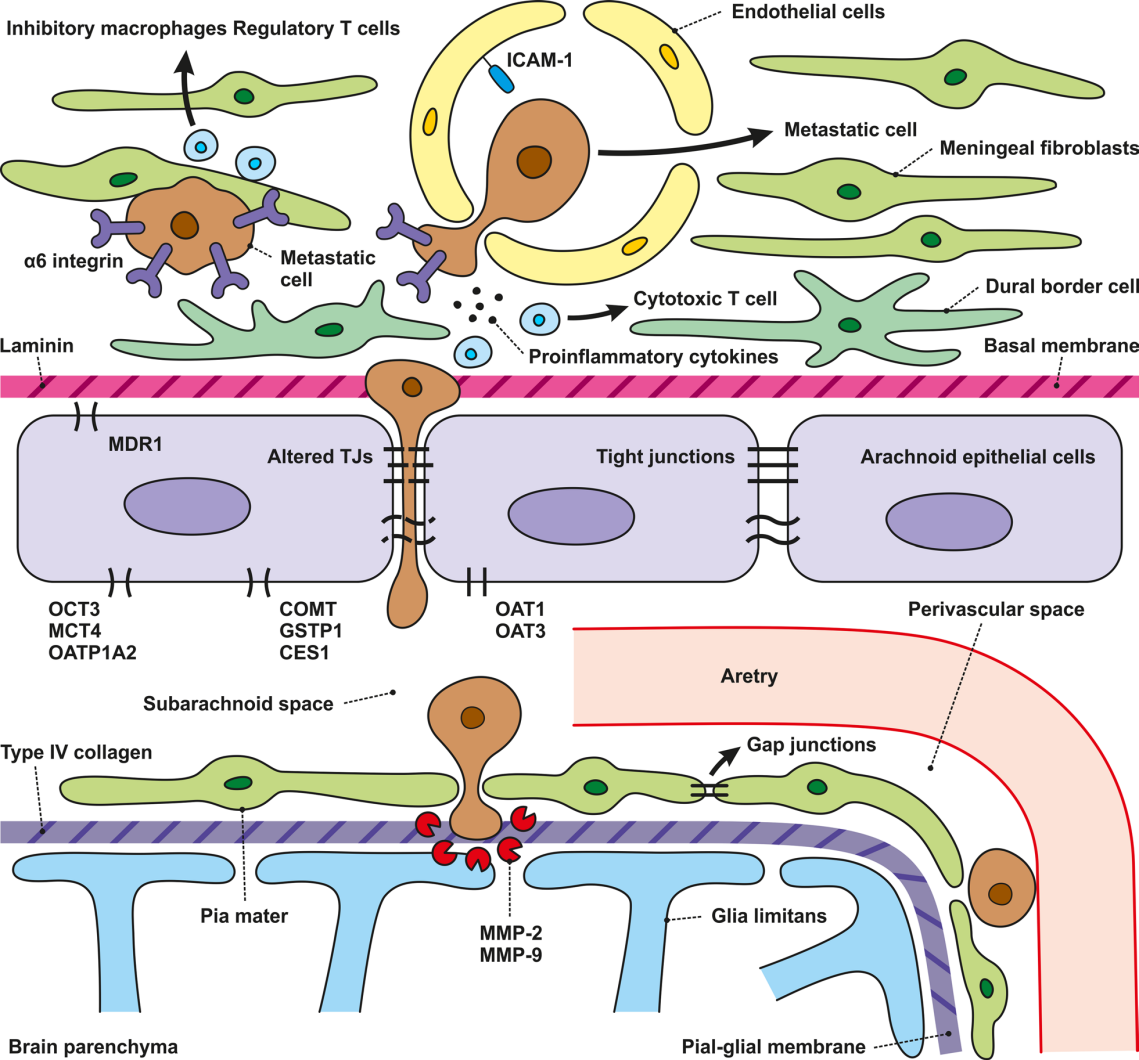
Schematic illustration showing transmigration of metastatic cells through the BAB into the subarachnoid space. Some metastatic cells, such as ALL lymphocytes, use α6 integrin to bind to laminin. After metastatic cells migrate to the meninges, surrounding immune cells release pro-inflammatory cytokines and recruit cytotoxic T cells. Later, metastatic cells begin to induce an immunosuppressive microenvironment including regulatory T cells (Tregs) and inhibitory macrophages. To reach the cerebrospinal fluid, metastatic cells must pass through the epithelial cells of the BAB, which are connected by TJ proteins. Metastatic cells in the subarachnoid space produce proteases such as MMP-2 and MMP-9 that degrade the pial-glial membrane containing type IV collagen and enter the brain tissue

The dura mater is considered an effective barrier against the spread of metastatic cells to the brain. Dural invasion is a less frequent route by which tumor cells spread to the CNS. However, it may occur secondary to lymphoma and adenocarcinoma of the stomach, breast, or lung [257]. In the literature, metastatic cell migration through the arachnoid and pia mater is referred to as leptomeningeal metastasis. The leptomeningeal system of tissues consists of the arachnoid mater, arachnoid villi, arachnoid vessels, arachnoid granulations, arachnoid trabeculae, cerebrospinal fluid, and pia mater [258]. The pia mater covers tunnels through blood vessels that pass through the CNS, known as perivascular spaces. The perivascular spaces are absent once blood vessels reach the capillary stage at the BBB. This layer is mainly composed of type IV collagen [259]. Ultrastructural studies have shown that the pia mater layer lining the brain vessels is fenestrated. Therefore, metastatic cells can move freely within leptomeninges [260] (Fig. 6). However, neoplastic cells also have the ability to disintegrate the leptomeningeal cell layer. This suggestion is supported by the finding of collagen fiber fragments in the gap between metastatic cells [261]. Metastatic cells use leptomeningeal blood vessels to enter the brain parenchyma. The migration of metastatic cells from the metastatic origin along leptomeningeal microvessels to the surrounding brain tissue contributes to the development of multiple brain lesions [262].

The basement membrane of leptomeningeal vessels is enriched in laminin. During acute lymphoblastic leukemia (ALL), lymphocytes use α6 integrin, a laminin receptor, to enter the leptomeninges and subarachnoid spaces. The arachnoid serves as a barrier that separates the CNS from the dura mater; however, lymphocytes are able to breach this barrier during ALL [263, 264]. Similarly, lymphocytes derived from B-cell lymphoma use the meninges or perivascular spaces to spread to the brain parenchyma. Neoplastic cells that form B-cell lymphomas present as solid meningeal tumors or diffuse tumors that spread in the brain along the leptomeninges.

B cells enter the perivascular space by rolling along the lumen of leptomeningeal blood vessels, followed by conformational changes in integrins, vessel formation, and migration through endothelial cells via diapedesis [265]. After metastatic cells reach the subarachnoid space, to infiltrate the brain, they must destroy the pia-glial membrane, which plays an important role in cerebroprotection. The pia-glial membrane is primarily composed of type IV collagen. It seems that the disintegration of the pial-glial membrane by metastatic cells occurs through type IV collagenases such as MMP-2 and MMP-9 [259].

The leptomeninges are sites of resident immune cells, including B cells, T cells, dendritic cells, and macrophages. When metastatic cells pass through the dura mater, leptomeningeal immune cells release pro-inflammatory cytokines and recruit cytotoxic T cells via adjacent lymphatic drainage. However, in the future, metastatic cells will start to propagate into immunosuppressive tumor microenvironments involving regulatory T cells (Tregs) and inhibitory macrophages. The immunosuppressive nature of metastatic cells may be reversed by immunotherapy, which reactivates the immune response [266].

### The circumventricular organs and brain metastasis

Circumventricular organs (CVOs) include secretory and sensory brain structures lacking the BBB. Secretory CVOs include the pineal gland, neurohypophysis (posterior pituitary), and median eminence. Sensory CVO includes the organum vasculosum of the lamina terminalis, the area postrema, and the subfornical organ [267]. Functionally, secretory CVOs that produce hormones and peptides are involved in neurochemical transport and chemoreception [268]. Moreover, sensory CVO controls chemicals from the circulatory system and CSF and conveys this information to neural effector centers to maintain body fluid homeostasis [267, 269]. A common feature of these organs is that they lack the blood–brain barrier (BBB). Therefore, substances in the blood can travel freely between fenestrated capillaries with loosely connected astrocytic endfeet and CVO tissues [267]. The CVO contains tenocytes, specialized ependymal cells with apical processes in contact with the CSF, and basal processes that contact fenestrated capillaries or neurons. These cells serve as gatekeepers; that is, they monitor the composition of the CSF and mediate this information to the CVO. Tanycytes are connected to tight junctions and communicate with each other through connexin 43 gap junctions [270, 271]. Owing to its rare occurrence, we will focus only on the most common sites of metastases in CVO, that is, the pineal and pituitary glands.

#### Pineal glands in relation to metastasis

Metastatic pineal gland lesions are uncommon. Owing to the absence of the BBB, hematogenous dissemination is the main route of metastasis to the pineal gland. According to autopsy reports, pineal metastasis is found in 0.4–3.8% of patients with solid tumors and in 5% of surgically treated patients with pineal tumors [271, 272]. In the literature, the most common types of primary tumors that metastasize to the pineal gland are lung, breast, kidney, esophagus, stomach, colon carcinoma, malignant melanoma, and myeloma [273, 274]. Metastatic lesions in the pineal gland are associated with leptomeningeal seeding through the CSF in 67% of cases [271].

### Metastatic spread in the sellar region

The sellar region, including the hypothalamic-pituitary axis, represents the coordinating center of the endocrine system. Metastases in the pituitary gland were found in 3% to 23% of cancer patients in an autopsy series [275]. The occurrence of clinical symptoms in patients with pituitary gland metastasis is uncommon, and most sellar metastases remain asymptomatic [276]. Lung and breast carcinomas are the most common primary tumors that metastasize to the pituitary gland [277, 278]. The hormonal environment may play a role in the development of pituitary gland metastasis, especially in breast cancer [279]. The absence of the BBB and high vascularity support a favorable niche for metastatic seeding [276]. Therefore, radiation sparing of the hypothalamic-pituitary area, which is highly radiosensitive, might be a treatment option for patients with a limited number of brain metastases not involving the sellar region [280]. This approach may prevent radiation-induced hormonal deficiencies.

### Blood-cerebrospinal fluid (B-CSF) barrier in relation to brain metastasis

**Fig. 7:**
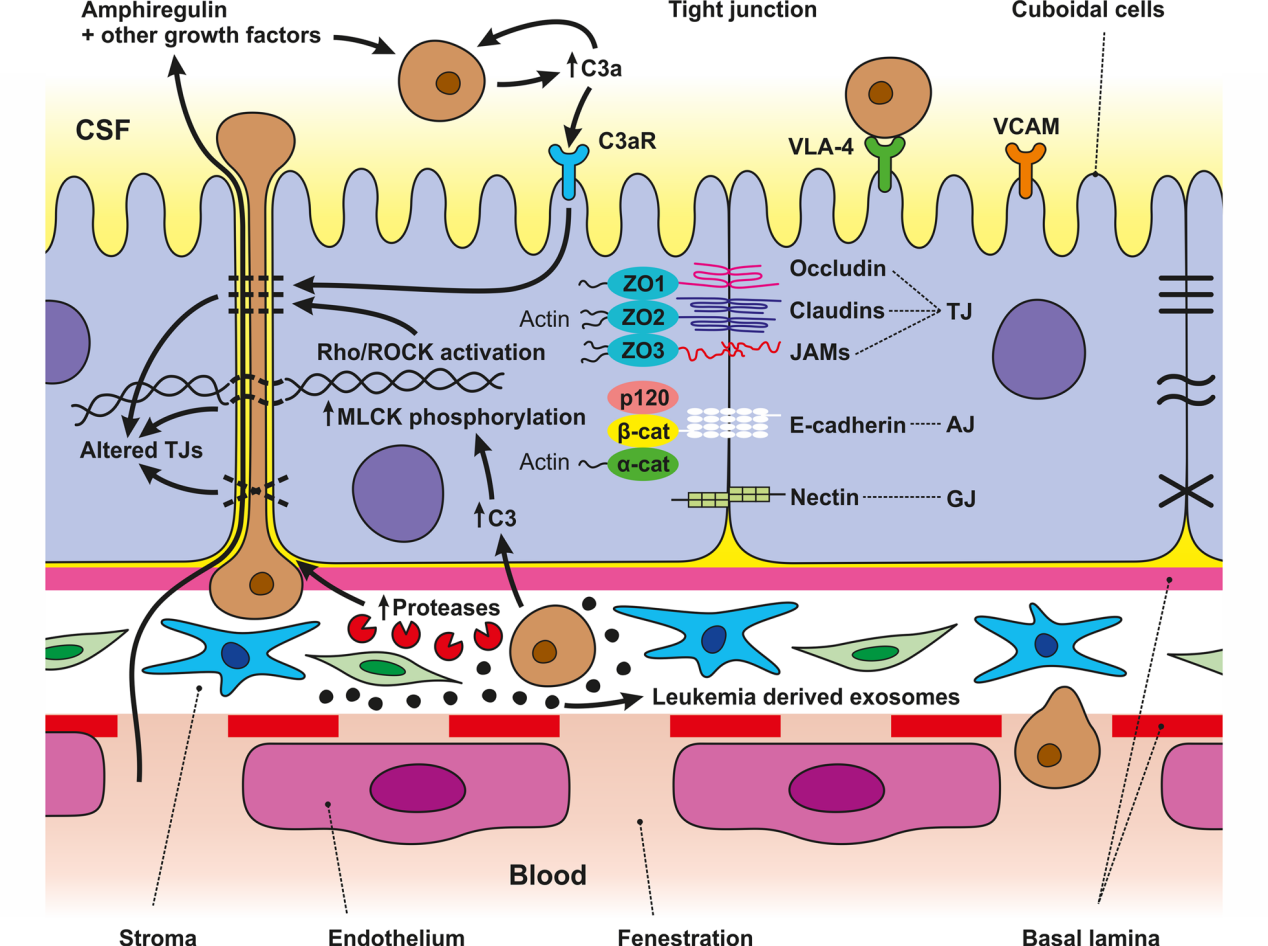
Invasion of metastatic cells through the B-CSF barrier Metastatic cells migrate through fenestrated capillaries into the CP, secrete proteases that degrade TJ proteins, and lead to B-CSF barrier damage. Additionally, myosin light-chain kinase (MLCK) phosphorylation, actin contraction via Rho/ROCK, and activation of the C3a-C3aR signaling cascade are involved in the alteration of TJ proteins. These changes in the B-CSF barrier potentiate the paracellular migration of metastatic cells and the transfer of growth factors into the central nervous system. The attraction and invasion of metastatic cells, especially ALL, is potentiated by VCAM-1 and VLA-4 localized on the luminal side of epithelial cells as well as leukemia-derived exosomes

Choroid plexus metastases (CPMs) are uncommon lesions and have generally been reported separately or in groups [218, 281, 282]. Due to the abundant vascular supply of the CP, the lateral ventricles are the most common sites of intraventricular metastases [283]. Under physiological conditions, the B-CSF barrier allows for immune surveillance of CSF. This concept is based on the permeability of the B-CSF barrier for cellular elements such as fluorescence-labeled T lymphocytes, which are found in the CP stroma and meninges after intravenous injection [284]. Leptomeningeal metastasis can develop from any solid tumor, but it often occurs in patients with lung or breast cancer [285]. The B-CSF barrier, as a potential entry point for leptomeningeal metastases, is often overlooked. A more permeable B-CSF barrier than the BBB predisposes this barrier to a higher rate of metastatic cell migration into the CNS. An in vitro study revealed that breast cancer cell migration across the B-CSF barrier was greater than that across the BBB. The loss of barrier properties in the presence of breast cancer may be associated with proteases secreted by cancer cells into the extracellular environment. This results in the degradation of TJ proteins between endothelial cells and, thus, in the degradation of the B-CSF barrier [286]. Transmigration of cancer cells through the layer of epithelial cells of the B-CSF barrier has been described in neuroblastoma, which can lead to leptomeningeal metastasis. After crossing fenestrated capillaries, neuroblastoma cells undergo paracellular transepithelial migration [287]. The exact mechanism by which cancer cells cross the B-CSF barrier is unknown, but alterations in TJ proteins through rearrangement of the actin cytoskeleton have been proposed (Fig. 7). This action is likely mediated by myosin-induced actin contraction via Rho/ROCK activation, resulting in TJ disassembly [288]. Similarly, leptomeningeal metastases produce C3, a central protein in the complement cascade that can affect the B-CSF barrier via myosin light-chain kinase (MLCK) phosphorylation. The main component of this cascade is C3a, an active molecule generated by the proteolysis of C3-by-C3 convertase. Alterations in the B-CSF barrier induced by C3a potentiate the entry of the EGFR ligand amphiregulin into the CSF, thus promoting the growth of leptomeningeal metastases [289]. While metastasis to the CP is generally rare, there have been multiple case reports documenting metastatic tumors in the CP of the lateral ventricle trigone, originating from a large-cell lung carcinoma [290, 291]. Moreover, an additional study indicated that CP metastasis from primary lung adenocarcinoma carrying the *EGFR* G719X mutation significantly improved following treatment with EGFR inhibitors [133].

The B-CSF barrier may play an essential role in regulating lymphocyte entry into the CNS [292–294]. Moreover, the selective permeability of the B-CSF barrier plays a role in the trafficking of leukemic cells into the CSF. Primary B-cell precursor (BCP) blasts of acute lymphoblastic leukemia (ALL) frequently infiltrate the CNS by crossing the B-CSF barrier rather than the BBB. Despite the finding that BCP-ALL cells express various chemokines that correspond to chemokine ligands expressed in B-CSF barrier cells, no clear evidence exists for the selection of leukemic cells bearing these receptors [295]. However, some integrins, such as very late antigen-4 (VLA-4) (α4:β1), which drives T-cell attraction to the CNS, are also expressed in the CP. Following migration through fenestrated capillaries and the CP epithelium, ALL cells adhere to VCAM-1 and VLA-4 (α4:β1), located on the apical side of epithelial cells [98, 296]. The invasion of leukemia cells across the B-CSF barrier into the CNS may be facilitated by leukemia-derived exosomes, which form the “pre-metastatic niche” and facilitate the invasion of circulating tumor cells into brain tissue. These small extracellular vesicles are actively produced and released by tumor cells in body fluids and modify healthy cells distant from the primary tumor site. There is some evidence that the invasion of ALL through the B-CSF barrier without destruction of barrier integrity may also be facilitated by these leukemia-derived exosomes [98]. The B-CSF barrier, as a primary transition route for ALL into the CNS, was supported by the finding of leptomeningeal infiltration of leukemic cells without parenchymal involvement [287].

## Conclusion

The BBB and B-CSF barrier remain the main brain barriers involved in the metastatic process. We have provided a complex review of many molecules involved in brain metastasis from lung cancer, breast cancer, and melanoma. The pathophysiological cascades involved in the migration of metastatic cells across brain barriers are complex and involve not only cellular but also non-cellular components. The major pathological processes involved in the transfer of metastatic cells through brain barriers include the upregulation of adhesion molecules, proteolytic enzymes, trophic factors, and neuroinflammatory changes.

On the basis of our review, little is known about the pathophysiological cascades and molecular mechanisms involved in the migration of metastatic cells into the CNS through the blood-arachnoid barriers. Interestingly, brain structures that lack the BBB, such as circumventricular organs, do not show metastases very often. This observation suggests the involvement of, protective mechanisms beyond the BBB, potentially including yet-undiscovered molecular components that regulate how tumor cells interact with those regions.

Since metastases are the most common type of brain tumor, a better understanding of how metastatic tumor cells use different mechanisms to cross the brain barrier may be a potential target for prevention and treatment. A deeper understanding of the pathophysiology of brain metastases will enable more frequent administration of targeted immunotherapy in personalized medicine approaches that could prevent or postpone whole-brain radiotherapy.

## Data Availability

No datasets were generated or analysed during the current study.

## Abbreviations

ADAM9: A disintegrin and metalloproteinase
AEG-1: Astrocyte elevated gene-1
AJ: Adherent junction
AKT: Ak strain transforming
ALK: Anaplastic lymphoma kinase
ALL: Acute lymphoblastic leukemia
Ang-2: Angiopoietin-2
ATPase: Adenosine triphosphate hydrolyzing enzymes
BAB: Blood-arachnoid barrier
BCRP: Breast cancer resistance protein
BCP: B-cell precursor
B-CSF: Blood-cerebrospinal fluid
BBB: Blood–brain barrier
BMs: Brain metastases
BSCB: Blood-spinal cord barrier
CADM2: Cell adhesion molecule 2
CAFs: Cancer-associated fibroblasts
CAMs: Cell adhesion molecules
CCL7: C–C Motif Chemokine Ligand 7
cGAMP: Cyclic GMP-AMP
CES1: Carboxylesterase 1
CNS: Central nervous system
COMT: Catechol-O-methyltransferase
CP: Choroid plexus
CPM: CP metastases
CTCs: Circulating tumor cells
CX3CR1: C-X3-C motif chemokine receptor 1
CXCR4: CXC chemokine receptor 4
CVO: Circumventricular organs
ECs: Endothelial cells
EGFR: Epidermal growth factor receptor
EMT: Endothelial-mesenchymal transition
ER: Estrogen receptor
ErbB2: V-erb-b2 avian erythroblastic leukemia viral oncogene homolog 2
EVs: Extracellular vesicles
FGF: Fibroblast growth factor
GJ: Gap junction
GLUT1: Glucose transporter 1
GSTP1: Glutathione-S-transferase protein 1
HER: Human epidermal growth factor receptor
HIF-1α: Hypoxia-inducible factor 1-alpha
HMMR: Hyaluronan-mediated motility receptor
HRAS: Harvey Rat sarcoma virus
ICAM-1: Intercellular adhesion molecule 1
IFN-γ: Interferon-gamma
IGFBP7: Insulin-like growth factor-binding protein 7
IL: Interleukin
INSR: Insulin receptor
JAK: Janus kinase
KRAS: Kirsten rat sarcoma virus
LOH: Loss of heterozygosity
LRP1: Lipoprotein receptor-related protein 1
Lamps: Lysosomal-associated membrane protein 1
MALAT1: Metastasis-associated lung adenocarcinoma transcript 1
MAPK: Mitogen-activated protein kinase
MBM: Melanoma BMs
MET: Mesenchymal-to-epithelial transition
MDR1: Multidrug resistance 1
miR: Micro RNA
MLCK: Myosin light-chain kinase
MMPs: Matrix metallopeptidases
MUC1: Mucin 1
NGF: Nerve growth factor
NK: Natural killer
NRAS: Neuroblastoma RAS viral oncogene homolog
NSCLC: Non-small cell lung cancer
NVU: Neurovascular unit
OAT: Organic anion transporters
PCDH7: Protocadherin 7
PET: Positron emission tomography
PI3K: Phosphatidylinositol-3 kinase
PLEKHA5: Pleckstrin homology domain-containing A5
PR: Progesterone receptor
PUMA: P53 upregulated modulator of apoptosis
RA: Reactive astrocytes
ROC: Receiver Operating Characteristics
SCLC: Small cell lung cancer
SIRT1: Silent mating type information regulation 2 homolog
SNPs: Single nucleotide polymorphisms
SOCS-1: Cytokine signaling-1
STAT3: Signal transducer and activator of transcription 3
ST6GALNAC5: ST6 N-Acetylgalactosaminide Alpha-2,6-Sialyltransferase 5
TDEs: Tumor-derived exosomes
TGF-β1: Transforming growth factor-beta 1
TIME: Tumor immune microenvironment
TIMP-1: Tissue inhibitor of metalloproteinases-1
TJs: Tight junctions
TK: Tyrosine kinase
tPA: Tissue-type plasminogen activator
TNBC: Triple-negative breast cancer
TNF-α: Tumor necrosis factor α
TP53: Tumor protein P53
VCAM-1: Vascular cell adhesion protein 1
VEGF: Vascular endothelial growth factor
VLA-4: Very late antigen-4
ZO: Zonula occludens proteins
